# Misregulation of Nucleoporins 98 and 96 leads to defects in protein synthesis that promote hallmarks of tumorigenesis

**DOI:** 10.1242/dmm.049234

**Published:** 2022-03-16

**Authors:** Ajai J. Pulianmackal, Kiriaki Kanakousaki, Kerry Flegel, Olga G. Grushko, Ella Gourley, Emily Rozich, Laura A. Buttitta

**Affiliations:** Molecular Cellular and Developmental Biology, University of Michigan, Ann Arbor, MI 48109, USA

**Keywords:** *Drosophila* wing, Nuclear pore complex, Ribosome biogenesis, JNK signaling, Apoptosis, Compensatory proliferation

## Abstract

Nucleoporin 98KD (Nup98) is a promiscuous translocation partner in hematological malignancies. Most disease models of Nup98 translocations involve ectopic expression of the fusion protein under study, leaving the endogenous *Nup98* loci unperturbed. Overlooked in these approaches is the loss of one copy of normal Nup98 in addition to the loss of Nup96 – a second Nucleoporin encoded within the same mRNA and reading frame as Nup98 – in translocations. Nup98 and Nup96 are also mutated in a number of other cancers, suggesting that their disruption is not limited to blood cancers. We found that reducing Nup98-96 function in *Drosophila melanogaster* (in which the Nup98-96 shared mRNA and reading frame is conserved) de-regulates the cell cycle. We found evidence of overproliferation in tissues with reduced Nup98-96, counteracted by elevated apoptosis and aberrant signaling associated with chronic wounding. Reducing Nup98-96 function led to defects in protein synthesis that triggered JNK signaling and contributed to hallmarks of tumorigenesis when apoptosis was inhibited. We suggest that partial loss of Nup98-96 function in translocations could de-regulate protein synthesis, leading to signaling that cooperates with other mutations to promote tumorigenesis.

## Abstract (precis)

**Editor's choice:** Compromising Nucleoporins 98 and 96 leads to defects in protein synthesis and stress signaling via JNK that triggers compensatory and apoptosis-induced proliferation.

## INTRODUCTION

Communication between the nucleus and cytoplasm occurs through nuclear pore complexes (NPCs), which are composed of highly conserved proteins termed Nucleoporins (Nups). Mutations in several Nups are associated with cancer, including loss-of-function mutations and translocations (Simon and Rout, 2014). Of the Nups associated with translocations, Nup98 is the most promiscuous (Lam and Aplan, 2001; Simon and Rout, 2014).

Nup98 function has been difficult to examine because the gene locus for Nup98 encodes two essential Nups, Nup98 and Nup96, which derive from an autocatalytic cleavage of a larger Nup98-96 polypeptide with Nup98 located at the amino terminus (Fontoura et al., 1999; Rosenblum and Blobel, 1999). However, a shorter *Nup98*-only transcript is also produced by the locus via alternative splicing (Fontoura et al., 1999). Nup98 is a peripheral Nup, found in nuclear pores and in the nucleoplasm (Griffis et al., 2002). It contains Phenylalanine-Glycine (FG) and GLFG repeats in its N-terminal region that allow Nup98 to interact with different nuclear transport receptors (Bachi et al., 2000; Moroianu et al., 1995) during nucleocytoplasmic shuttling, and it has a role in regulating gene transcription (Capelson et al., 2010; Kalverda et al., 2010). In contrast, Nup96 is a core scaffold protein; it is stably localized at the NPC and is part of the core Nup107-160 complex (Walther et al., 2003).

All Nup98 chromosomal translocations that have been observed have a breakpoint in the 3′ end of the Nup98 portion, disrupting the Nup98 coding region located upstream of Nup96 (Xu and Powers, 2009). Thus, Nup98 translocations result in fusions of the N-terminal region of Nup98 with the C-terminal region of a partner gene, which varies (Simon and Rout, 2014). This almost certainly disrupts the expression of Nup96 as well, which requires Nup98-dependent autocatalytic processing from the Nup98-96 precursor protein to be properly localized and functional (Fontoura et al., 1999; Rosenblum and Blobel, 1999).

Although most of the attention on Nup98 translocations in cancer has focused on overexpressing the fusion partners, there is increasing evidence that the disruption of endogenous Nup98 and/or Nup96 may contribute to enhanced proliferation that could cooperate with other oncogenic mutations. Mice carrying a stop codon knocked into the 3′ end of the Nup98 portion of the shared *Nup98-96* transcript have been used to examine loss of Nup96 function in the presence of intact Nup98 protein (Faria et al., 2006). Loss of one copy of Nup96 in the mouse leads to mildly enhanced proliferation of T-cells, supporting a potential role for Nup96 as a haplo-insufficient tumor suppressor (Chakraborty et al., 2008), but *Nup96*^+/−^ mice do not appear to exhibit cell cycle deregulation in other tissues or develop cancer (Faria et al., 2006). Conversely, an engineered allele generating loss of one copy of *Nup98* in the mouse, but with Nup96 protein expression remaining intact, cooperates with loss of the nuclear export cofactor Rae1 to increase aneuploidy (Jeganathan et al., 2005), but *Nup98*^+/−^ mice have not been reported to develop cancer, nor to exhibit cell cycle de-regulation on their own (Wu et al., 2001). Studies of *Nup98* and *Nup96* homozygous mutants have been severely limited by the very early embryonic lethality caused the by loss of each Nup (Faria et al., 2006; Wu et al., 2001), and compound mutants have not been reported. Using a small interfering RNA (siRNA) knockdown approach to selectively target Nup98 in human cells revealed a role for Nup98 in p53-dependent induction of the Cdk inhibitor p21 in response to DNA damage, consistent with a tumor-suppressor function for Nup98 (Singer et al., 2012).

Work in *Drosophila* revealed an unexpected off-pore role for Nup98 in modulating the expression of several cell cycle genes (Capelson et al., 2010; Kalverda et al., 2010). Loss of Nup98-96 function in *Drosophila* is lethal and pleiotropic. Flies homozygous for an allele with a stop codon predicted to generate a truncated Nup98 and eliminate Nup96 die prior to metamorphosis (Parrott et al., 2011; Presgraves et al., 2003). A *Nup98-96* allele disrupted by a transposon insertion in the fourth exon of *Nup98*, predicted to disrupt splicing, exhibits germline-specific defects in stem cell proliferation and differentiation (Parrott et al., 2011). Low-level constitutive depletion of Nup98-96 by RNA interference (RNAi) in adult flies impacts expression of anti-viral genes (Panda et al., 2014), while acute inhibition of Nup98-96 in imaginal discs leads to misregulation of Hox gene expression (Pascual-Garcia et al., 2014). Consistent with pleiotropic effects, the knockdown of Nup98-96 by RNAi has emerged in a number of screens in *Drosophila*, revealing roles in nuclear translocation of specific proteins (Dopie et al., 2015; Kristo et al., 2017), and blood progenitor proliferation and differentiation (Mondal et al., 2014).

Human *NUP98-96* (also known as *NUP98*) is located near a known imprinted tumor-suppressor region in the genome (Joyce and Schofield, 1998), which could be significant as loss of heterozygosity via mutation or epigenetic modifications for the remaining *NUP98-96* locus may occur in cancers exhibiting translocations. We are not aware of any information reported to date about the expression levels from the non-translocated *NUP98-96* gene in these diseases. We simultaneously inhibited Nup98 and Nup96 in *Drosophila* using an *in vivo* RNAi knockdown approach and observed cell cycle de-regulation and cooperation with oncogenic mutations, consistent with a tumor-suppressor function for Nup98 and/or Nup96. Transgenes encoding Nup98 or Nup96 individually do not rescue this phenotype, while expression of a transgene encoding both does, suggesting that Nup98 and Nup96 play non-overlapping and potentially synergistic roles in cell cycle regulation.

Here, we show that that reducing Nup98-96 function via an RNAi approach in *Drosophila melanogaster* (in which the Nup98-96 shared mRNA and reading frame gene structure is conserved) de-regulates the cell cycle. We find evidence of overproliferation in Nup98-96-deficient tissues, counteracted by elevated apoptosis and aberrant JNK signaling associated with wound healing. When the knockdown of Nup98-96 is combined with inhibition of apoptosis, we see synergism leading to overgrowth consistent with a tumor-suppressor function for endogenous Nup98 and/or 96. We suggest that the loss of normal Nup98 and Nup96 function may de-regulate the cell cycle to cooperate with other mutations in cancer.

## RESULTS

### Loss of Nup98-96 disrupts G1 arrests and causes cell cycle de-regulation

We previously described an RNAi screen to identify genes that promote proper cell cycle exit in *Drosophila* eye (Flegel et al., 2016; Sun and Buttitta, 2015). Our initial screen used UAS-RNAi constructs from the Harvard TRiP RNAi collection, driven by the *Glass Multimer Repeats* (*GMR*) promoter-Gal4 with an E2F-responsive *PCNA-white* reporter transgene, which provides adult eye color as a readout of E2F and cell cycle activity (Bandura et al., 2013). This screen successfully identified genes that delay proper cell cycle exit by promoting a delay or bypass of G1 arrest, which directly or indirectly impacts E2F activity (Flegel et al., 2016; Sun and Buttitta, 2015). In this screen, we identified an RNAi line targeting the bi-cistronic *Nup98-96* transcript as a potential novel regulator of cell cycle exit in the *Drosophila* eye.

Cell cycle exit in the eye is normally completed by 24 h after puparium formation (APF). To confirm whether knockdown of *Nup98-96* delayed cell cycle exit in the pupa eye, we performed S-phase labeling via 5-ethynyl-2′-deoxyuridine (EdU) incorporation and examined an E2F transcriptional activity reporter *PCNA-GFP* in pupal eyes several hours after normal cell cycle exit. We confirmed that knockdown of *Nup98-96* delayed proper cell cycle exit in the pupa eye to between 28 h and 36 h APF (Fig. S1A). We also confirmed that the RNAi line identified in the screen knocked down endogenous Nup98-96 protein tagged with GFP and that re-expression of both exogenous *Nup98* and *Nup96* was required to rescue phenotypes due to *Nup98-96* bi-cistronic transcript knockdown (Fig. S1B,C). Neither exogenous *Nup98* nor *Nup96* alone was sufficient to rescue *Nup98-96* RNAi phenotypes, suggesting that both Nups contribute to the cell cycle exit defect.

**Fig. 1.**
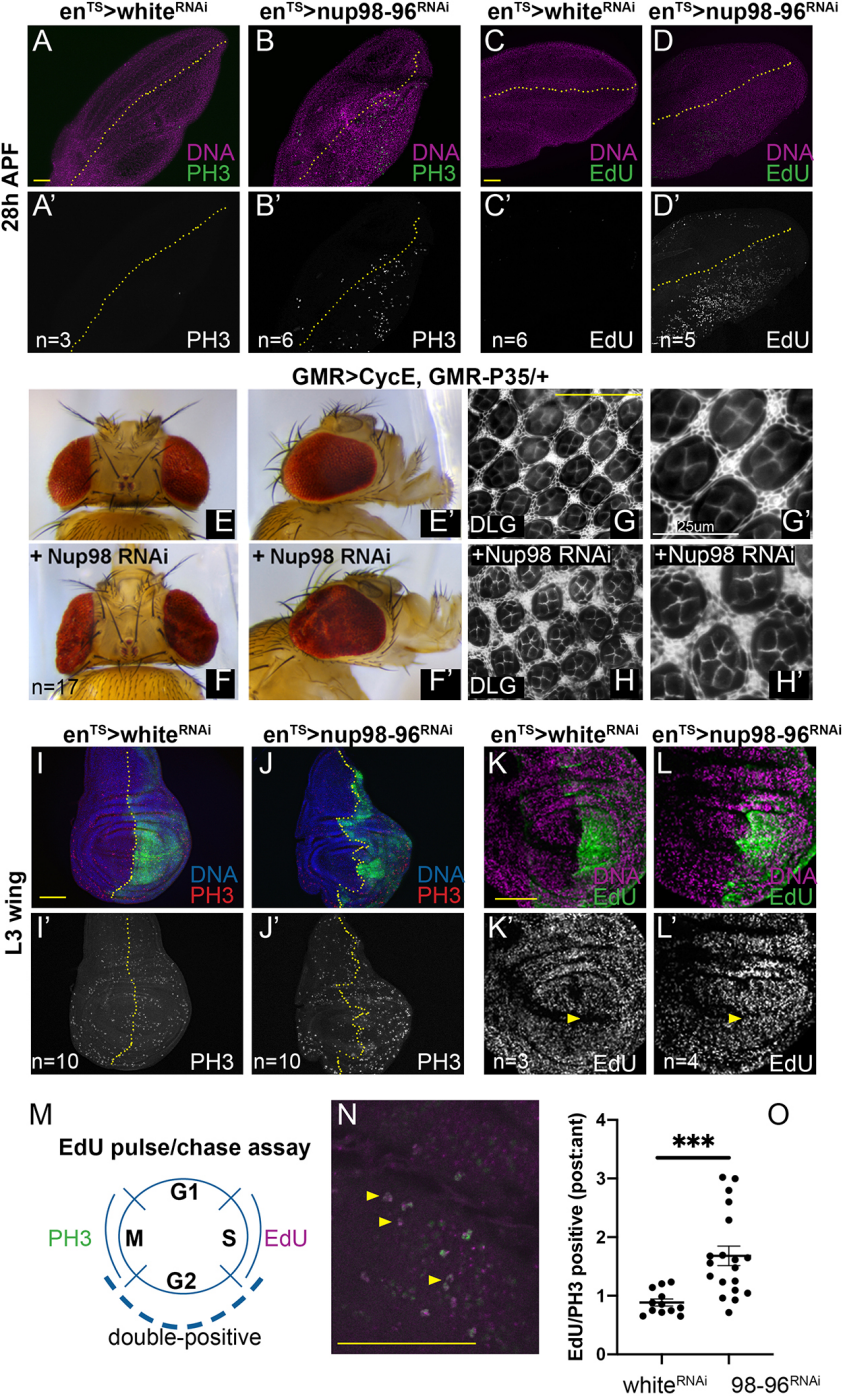
**Inhibition of Nup98-96 leads to G1 bypass and cell cycle de-regulation.** (A-D′) Using *engrailed*-*Gal4* modified with a temperature-sensitive Gal80 (*en^TS^*), the indicated UAS-RNAis were expressed in the posterior wing disc from mid-L3 to 28 h after puparium formation (APF) at 28°C. The dotted lines indicate the pupal wing anterior–posterior (A-P) boundary. Panels are shown in single color in A′, B′, C′ and D′ and in similar panels in this and other figures. Nup98-96 inhibition increased the number of mitoses (indicated by phospho-Ser10 histone H3, PH3) and S phases [indicated by 5-ethynyl-2-deoxyuridine (EdU) labeling in the posterior wing], at stages when the wing is normally post-mitotic. (E,E′) Adult eyes from a heterozygous sensitized background expressing *UAS-cyclin E* (*CycE*) under the *GMR*-*Gal4* promoter and *GMR*-driven *P35* are shown. (F-H′) Adding in *UAS-Nup98-96* RNAi enhanced eye size and folding (F,F′), and increased the number of cone cells and interommatidial cells, as shown by staining for the septate junction protein Discs large (Dlg; also known as Dlg1) (G-H′). (I-L′) Using *en^TS^*, the indicated *UAS*-RNAis were expressed in the posterior wing disc for 72 h prior to dissection of wandering L3 larvae. The dotted lines indicate the A-P boundary. Nup98-96 inhibition increased the number of mitoses and S phases in the posterior wing disc. The EdU experiment was performed multiple times with 5, 10 or 20 min of EdU labeling. Data and number of replicates from 5 min of EdU labeling are shown. Yellow arrowheads in K′ and L′ indicate the posterior zone of non-proliferating cells (ZNC), which is normally G1 arrested, but undergoes S phases when Nup98-96 is knocked down. (M) An EdU pulse for 1 h followed by a 7 h chase and PH3 staining was used to label mid-L3 wing disc cells that progress from S to M phase in ∼8 h. This experiment was repeated three times, with intervals of 6, 7 and 8 h chase. (N) Examples of PH3 (green)/EdU (magenta) double-labeled cells are shown (yellow arrowheads). (O) Quantification of double-labeled cells in the posterior:anterior compartments normalizes for EdU incorporation in each disc and provides an indication of cell cycling speed differences between compartments. RNAi to Nup98-96 increased cycling speed in the posterior wing disc (***P<0.024; *t*-test with Welch's correction). Plots of individual biological replicates include mean±s.e.m. Yellow scale bars: 50 µm; white scale bar: 25 μm.

We next examined whether knockdown of *Nup98-96* in the posterior wing using the driver *engrailed-Gal4* (*en-Gal4*) with a temperature-sensitive Gal80 (*en^TS^*) could delay cell cycle exit in the pupal wing, which also completes the final cell cycle by 24 h APF. We used *Gal80^TS^* to limit expression of the RNAi to pupal stages to avoid developmental delays and lethality, and an RNAi to the eye pigment gene *white* (*white^RNAi^*), which has no effect on cell cycle exit served as a negative control (Flegel et al., 2016). Labeling S phases with EdU incorporation from 26 h to 28 h APF and mitoses using anti-phosphorylated Ser10-Histone H3 (PH3) antibody revealed that knockdown of *Nup98-96* delayed cell cycle exit in the wing until 28-30 h APF (Fig. 1A-D′).

We have shown that delays in cell cycle exit accompanied by high E2F activity can result from slowing the final cell cycle, or by causing additional cell cycles (Flegel et al., 2016; Sun and Buttitta, 2015). To determine which is the case with knockdown of *Nup98-96*, we expressed *Nup98-96* RNAi in the eye, using a sensitized background with the *GMR*-Gal4 driver driving the G1-S Cyclin, Cyclin E (*CycE*) and the baculoviral apoptosis inhibitor *P35* (Hay et al., 1994). This sensitized background causes enlarged eyes and one to three extra cell cycles in the pupa eye prior to a robust cell cycle exit (Sun and Buttitta, 2015). The enlarged eyes of this sensitized background are visibly suppressed by factors that delay the cell cycle and enhanced by manipulations that cause extra cell cycles (Sun and Buttitta, 2015). Knockdown of *Nup98-96* effectively enhanced the eye overgrowth of this sensitized background and resulted in extra cone cells and extra interommatidial cells in the pupal eye, confirming that the delay of cell cycle exit was caused by additional cell cycles (Fig. 1E-H′).

We next examined proliferating larval wing discs to determine whether the effects of *Nup98-96* knockdown were specific to the pupa or also impacted earlier cell cycles. We used *en-Gal4/Gal80^TS^* to express *Nup98-96* RNAi in the posterior wing disc, labeled with GFP, for 72 h prior to dissection, and detected mitoses with PH3 or performed 5-10 min of EdU labeling for S phase immediately prior to fixation. We observed an increase in mitoses when *Nup98-96* was knocked down, accompanied by an increase in S-phase labeling (Fig. 1I-L′; Fig. S1F). Consistent with knockdown of *Nup98-96* leading to a bypass of a G1 cell cycle arrest, we also observed abundant S phases in the posterior zone of non-proliferating cells (ZNC) (Fig. 1K′,L′, yellow arrowheads), which are normally quiescent at this stage (Johnston and Edgar, 1998). Similar effects on larval wing disc proliferation were also observed using two independent *Nup98-96* RNAi lines from the Vienna *Drosophila* Resource Center (VDRC) collection (Fig. S1D).

Increased EdU and PH3 labeling at fixed time points can be due to increased proliferation or increased time spent in S and M phases, respectively. To examine whether S to M progression is altered when *Nup98-96* is knocked down, we performed an EdU pulse-chase assay combined with PH3 labeling in L3 larval wing discs. We fed larvae with food containing EdU for 1 h followed by a chase without EdU for 7 h. At the end of the chase, we fixed larval wing discs and stained for PH3 and scored the number of mitotic cells double positive for EdU and PH3 in the posterior versus anterior wing pouch for *white* RNAi versus *Nup98-96* RNAi discs. The posterior to anterior ratio of double-positive cells that transition from S to M phase in control *white* RNAi discs is ∼1, indicating similar cell cycle timing in the posterior and anterior wing disc of late L3 larvae (Mesquita et al., 2010). By contrast, the fraction of EdU-positive mitoses in the posterior compared to the anterior disc was increased when *Nup98-96* was knocked down in the posterior, suggesting that more of these cells are progressing from S to M within 7 h (Fig. 1M-O). An increased posterior to anterior ratio could indicate either an increase in proliferation rate in the posterior disc, or a non-autonomous decrease in the anterior (Mesquita et al., 2010). Indeed, the increased ratio of EdU-positive mitoses in the *Nup98-96* RNAi domain is, in part, due to a non-autonomous effect, resulting in fewer S-M transitions in 7 h in the anterior compartment with the *Nup98-96* knockdown (Fig. S1G). However, when we compare the fraction of EdU-positive mitoses in *Nup98-96* RNAi posterior discs to posterior *white* RNAi wings (an external control), we observe a ∼20% average increase in EdU^+^ mitoses, although it is not statistically significant. Altogether, we conclude that cells with *Nup98-96* knocked down proliferate faster than their neighbors and proliferate at rates similar to or slightly faster than control cells.

### *Nup98-96* knockdown results in apoptosis and activation of JNK signaling

**Fig. 2.**
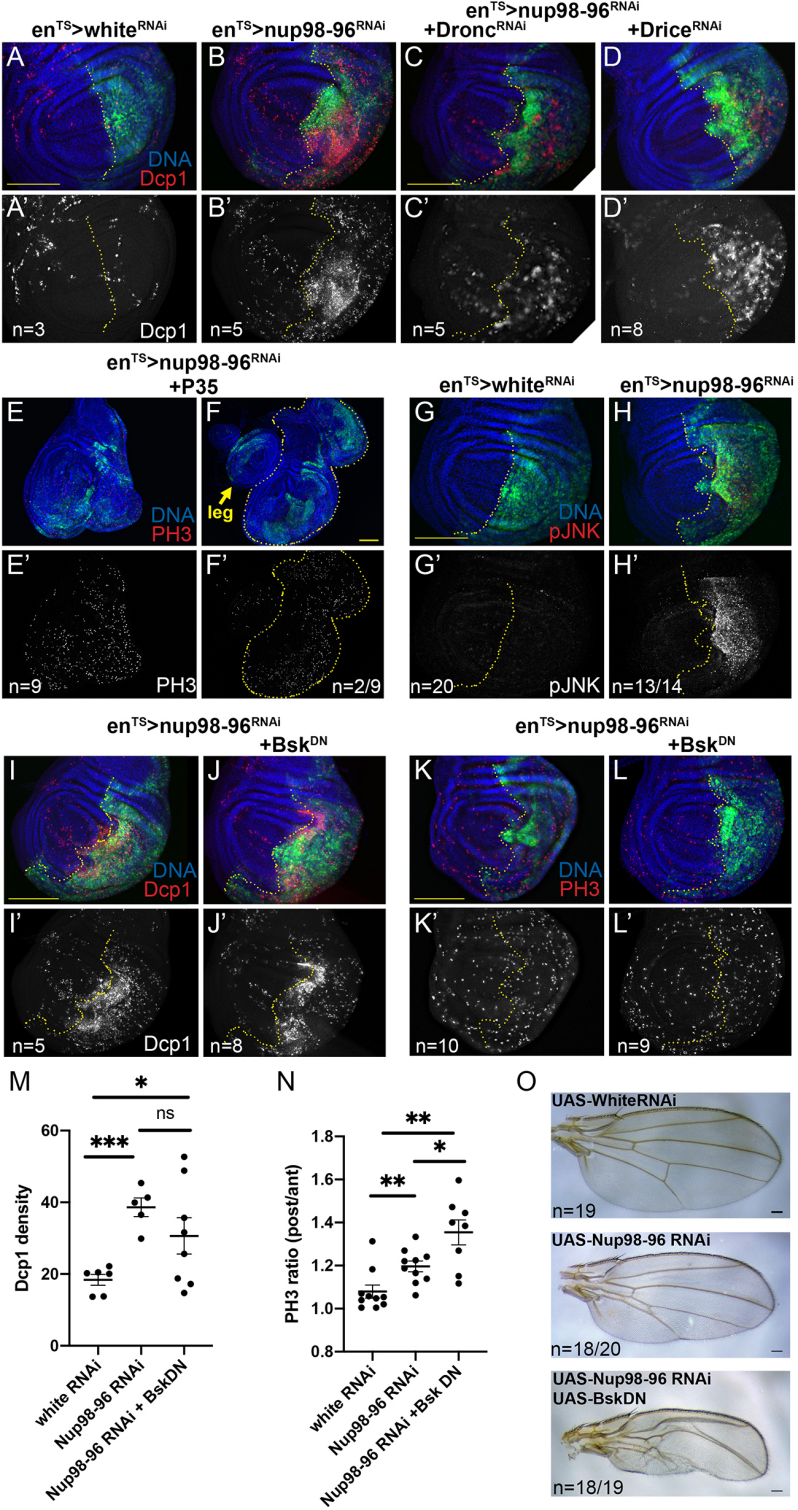
**Inhibition of Nup98-96 leads to cell death and compensatory proliferation.** (A-L′) Using *en^TS^*, the indicated *UAS*-RNAis were expressed in the posterior wing disc for 72 h prior to dissection of wandering L3 larvae (unless otherwise indicated). The dotted lines indicate the A-P boundary. (A-D′) Nup98-96 inhibition increased apoptosis in the posterior disc, as indicated by cleaved Death caspase-1 (DCP1). (E-F′) Co-expression of *UAS-P35* with *Nup98-96* RNAi led to tissue overgrowth (E,E′) and, by day 5, wing pouch duplication, outlined in yellow (F,F′). (G-H′) Nup-98-96 knockdown led to activation of JNK signaling as detected by phosphorylated JNK staining (pJNK). (I-N) Co-expression of a dominant-negative form of *Drosophila* JNK, *Basket* (*Bsk^DN^*) had variable effects on DCP1 staining and increased the ratio of PH3 labeling in posterior:anterior discs, although overall PH3 signal decreased with *Bsk^DN^* (Fig. S2) (ns, not significant; *P<0.05, **P<0.01, ***P<0.005; Welch's *t*-test comparisons). (O) Adult wings expressing the indicated transgenes with *en^TS^*. Co-expression of *Bsk^DN^* with *Nup98-96* RNAi severely reduced the size of the posterior wing. Plots of individual biological replicates include mean±s.e.m. Scale bars: 100 µm.

Despite the increased proliferation and disruption of G1 arrest in the larval and pupal tissues, we noted that the posterior wing expressing *Nup98-96* RNAi was consistently smaller than normal, suggesting an increase in cell death (Fig. S1C). Indeed, knockdown of *Nup98-96* for 72 h dramatically increased apoptosis in the posterior wing disc, as measured by anti-cleaved Caspase 3 and anti-*Drosophila* Caspase 1 (DCP1) staining (Fig. 2A-B′; Fig. S2A-I″). The increased apoptosis and reduced size in the posterior disc could be fully rescued by exogenous expression of both *Nup98* and *Nup96* in the presence of *Nup98-96* RNAi (Fig. S1C, Fig. S2C,D). Expression of *Nup98-96* RNAi in the dorsal wing disc using *apterous-Gal4,Gal80^TS^* (*ap^TS^*) for 72 h also induced robust apoptosis, indicating that the effect was not specific to the posterior disc (Fig. S2E). We knocked down the initiator caspase Dronc or effector caspase Drice in an attempt to rescue the apoptotic cells, but neither fully suppressed the apoptotic response to *Nup98-96* knockdown (Fig. 2C,D), nor did co-expression of a dominant-negative form of p53 (Fig. S2F; Brodsky et al., 2000). We next co-expressed the baculoviral caspase inhibitor P35 with *Nup98-96* RNAi, which suppressed apoptosis (Fig. S2G-I) and resulted in dramatic wing disc overgrowth phenotypes, including folding of the epithelium and occasional duplication of wings (Fig. 2E,F). The overgrowth and duplication of wing tissues was reminiscent of a phenotype observed during wing damage and regeneration when JNK signaling is activated (Perez-Garijo et al., 2009; Schuster and Smith-Bolton, 2015; Verghese and Su, 2017; Worley et al., 2018). We therefore examined whether *Nup98-96* knockdown resulted in activation of JNK signaling by staining for phospho-JNK (pJNK) (Fig. 2G,H) and induction of the JNK signaling transcriptional target *puckered* (using a *puc-LacZ* expression reporter; Fig. S2J). Knockdown of *Nup98-96* for 72 h led to high levels of compartment-autonomous JNK signaling in the wing disc.

High JNK signaling can paradoxically lead to both proliferation and cell death in *Drosophila* tissues (Fogarty and Bergmann, 2017). We next tested whether inhibition of JNK signaling via a dominant-negative form of the *Drosophila* JNK, *Basket* (*Bsk^DN^*), could suppress the apoptotic and proliferative response to knockdown of *Nup98-96*. Co-expression of *Bsk^DN^* with *Nup98-96* RNAi had a complex effect on apoptosis in the wing, enhancing levels of apoptosis in some samples, while suppressing in others (Fig. 2I-J′,M). Unexpectedly, co-expression of *Bsk^DN^* with *Nup98-96* RNAi did not suppress the increased mitoses observed in posterior wings expressing *Nup98-96* RNAi, and even mildly enhanced the differences in mitotic labeling between anterior and posterior compartments (Fig. 2K-L′,N). Although, we noted an overall decrease in PH3 labeling across both compartments when *Bsk^DN^* was co-expressed in the posterior wing disc (Fig. S2K), suggesting that blocking JNK signaling reduced compensatory proliferation both autonomously and non-autonomously. The few adult wings that could be recovered with both *Nup98-96* RNAi and *Bsk^DN^* expression exhibited a more severely reduced posterior compartment than with *Nup98-96* RNAi alone (Fig. 2O). This suggests that activation of JNK signaling provides compensatory proliferation and may partially increase survival when *Nup98-96* is knocked down, consistent with previously described roles in wing damage and regeneration (Bergantinos et al., 2010; Herrera et al., 2013).

### *Nup98-96* knockdown leads to mispatterning and gene expression resembling a wound-healing and loser phenotype

The JNK signaling and overgrowth phenotypes caused by suppressing apoptosis during *Nup98-96* knockdown are reminiscent of a phenomenon called apoptosis-induced compensatory proliferation (AIP) (Fogarty and Bergmann, 2017), which can impact tissue patterning. As previously described for other JNK-driven *Drosophila* tumor models, we observed dramatic tissue folding and invasion behaviors at both the anterior–posterior (A-P) and dorsal–ventral (D-V) compartment boundaries when *Nup98-96* was inhibited in the presence of *P35* expression (Fig. S3A-C) (Muzzopappa et al., 2017). Therefore, we next investigated whether wing disc patterning is disrupted by *Nup98-96* knockdown, as previously shown in AIP.

**Fig. 3.**
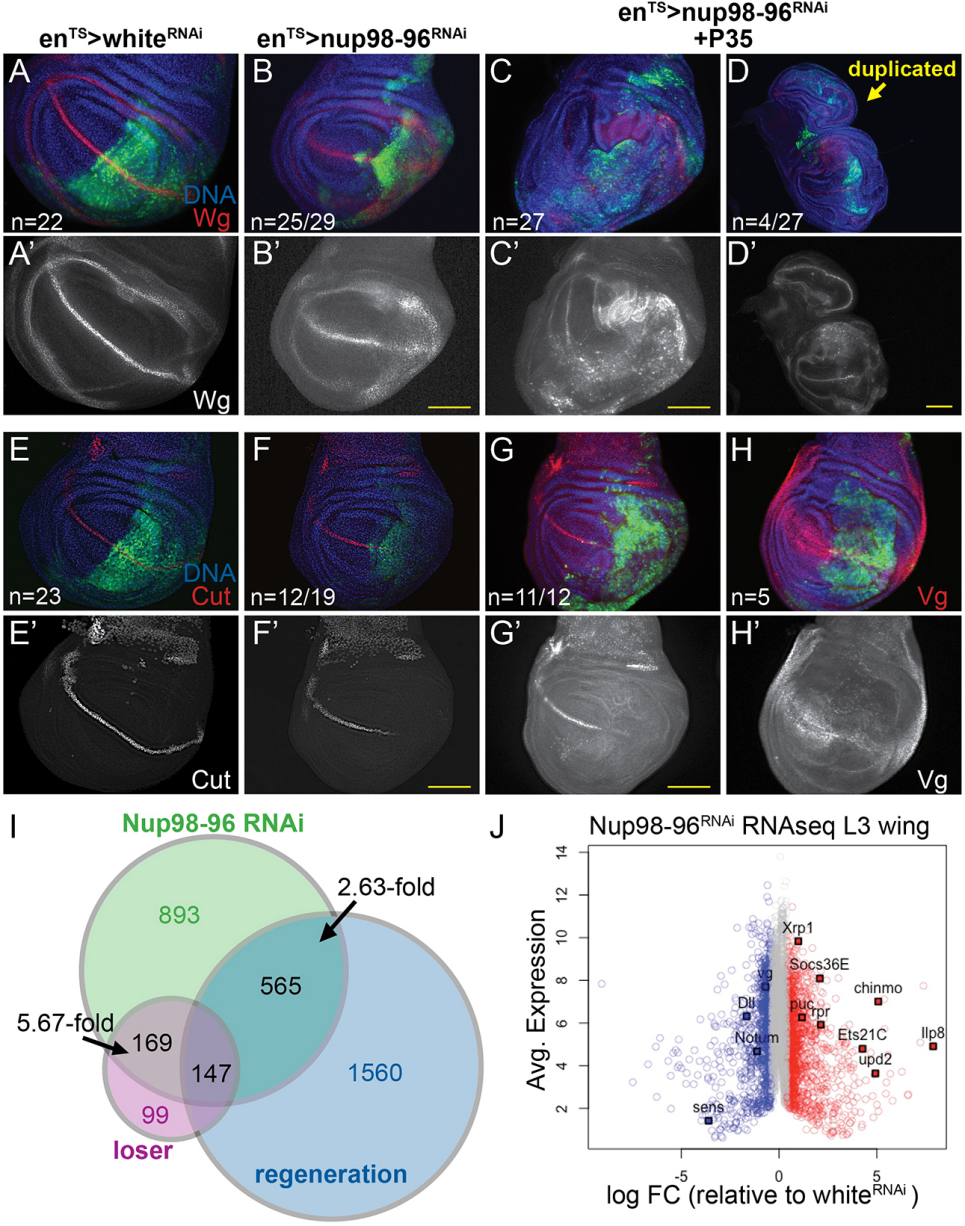
**Inhibition of Nup98-96 leads to mispatterning, gene expression changes associated with wounding and a ‘loser’ phenotype.** (A-H′) Using *en^TS^*, the indicated *UAS*-RNAis were expressed in the posterior wing disc for 72 h prior to dissection of wandering L3 larvae (unless otherwise indicated). Discs in C, D, G and H co-express P35 to block apoptosis and allow for tissue overgrowth. Samples in C and D were dissected after 5 days of *Nup98-96* RNAi+*P35* expression. (A-D′) Wg levels are disrupted at the dorsal–ventral (D-V) margin but increased at the dorsal hinge upon *Nup98-96* knockdown. The effect on Wg and wing disc overgrowth is enhanced by P35. (E-G′) Cut expression at the D-V margin is disrupted by *Nup98-96* knockdown, independent of P35 expression. (H,H′) Vestigial (Vg) is reduced when Nup98-96 is knocked down. (I,J) RNAseq was performed on dissected late L3 wing discs expressing *UAS-Nup98-96* or *white* RNAi for 72 h, driven by *apterous-*Gal4 with *tub-Gal80^TS^* (*ap^TS^*). (I) A comparison of the overlap of genes significantly altered by *Nup98-96* RNAi (0.5-log_2_fold or more) to previously published ‘wounding’ and ‘loser’ gene expression signatures in wings. The fold enrichment in the overlap of genes above that expected by chance is shown. (J) An M-A plot of the RNAseq data with significantly increased expression indicated in red and significantly decreased expression in blue. Genes in gray are not significantly altered. Scale bars: 100 µm.

We first examined Wg levels in discs expressing *Nup98-96* RNAi, because AIP and wing duplications have been associated with ectopic Wg (Baonza et al., 2000; Perez-Garijo et al., 2009; Verghese and Su, 2017; Worley et al., 2018). We found that knockdown of *Nup98-96* resulted in ectopic Wg in the dorsal wing hinge, and this effect was amplified in the presence of P35 (Fig. 3A-D′). We also observed ectopic phosphorylation of the transcription factor Mad (Fig. S3D), consistent with the previously described effect of AIP on Dpp signaling (Perez-Garijo et al., 2009; Pinal et al., 2018).

Both Wg and Notch have been implicated in G1 arrest in the posterior ZNC (Duman-Scheel et al., 2004; Herranz et al., 2008). We therefore next examined the expression of two targets of Notch and Wg signaling: Cut, which is expressed in G1-arrested cells at the D-V boundary, and Vestigial (Vg), which is expressed in a broader domain of the pouch induced by longer-range Wg signaling (de Celis et al., 1996; Kim et al., 1996; Neumann and Cohen, 1997). We found that Cut expression at the D-V boundary was nearly eliminated when *Nup98-96* was knocked down, both with and without *P35* (Fig. 3E-G′). This suggests that Notch signaling at the D-V boundary is compromised when *Nup98-96* function is reduced. Vg, an important wing identity and growth regulator (Halder et al., 1998; Williams et al., 1991, 1993; Zecca and Struhl, 2010), was also dramatically reduced in the pouch upon *Nup98-96* knockdown (Fig. 3H,H′), suggesting that Wg released from the D-V boundary is also compromised. Notch and Wg have been suggested to regulate the ZNC cell cycle arrest via repression of dMyc (also known as Myc) expression, but we did not observe any effects of *Nup98-96* knockdown on dMyc levels in the ZNC. Interestingly, the downregulation of Vg was also observed in regenerating discs (Smith-Bolton et al., 2009), potentially due to the replacement of dying pouch cells with cells from the neighboring areas of the wing (Zecca and Struhl, 2010). Taken together, these data demonstrate that reduction of Nup98-96 function in the presence of P35 leads to AIP and wing mispatterning and cell identity changes associated with a chronic wounding and regeneration response.

Although high JNK signaling and AIP can explain many of the phenotypes we observe with *Nup98-96* knockdown, this does not reveal the proximal defect caused by loss of Nup98-96 function. To determine additional effects of *Nup98-96* knockdown on gene expression in the wing, we performed comparative gene expression analysis via RNA sequencing (RNAseq) to identify mRNAs increased or decreased upon *Nup98-96* RNAi compared to the control *white* RNAi for 72 h in late L3 wing discs (Table S1). We observed the strong upregulation of many genes directly associated with JNK signaling (e.g. *puc*, *Mmp1*, *Ets21C*) (Kulshammer et al., 2015; McEwen and Peifer, 2005; Uhlirova and Bohmann, 2006), JAK/STAT signaling [*upd* (also known as *upd1*), *upd2*, *Socs36E*] (Amoyel et al., 2014) and developmental delays associated with wing damage and regeneration (*chinmo*, *Ilp8*) (Colombani et al., 2012; Garelli et al., 2012; Katsuyama et al., 2015; Narbonne-Reveau and Maurange, 2019). Consistent with the wing overgrowth phenotypes, several of the genes listed above have been shown to act in combination to promote tumorigenic overgrowth in flies (Toggweiler et al., 2016), and we see a striking overlap of about one-third of the genes changed upon *Nup98-96* RNAi with gene expression changes observed in a well-established invasive fly tumor model (507 of 1774 genes; Table S1) (Kulshammer et al., 2015).

Consistent with increased proliferation, we also observed the upregulation of several DNA damage and replication genes regulated by E2F activity (*Orc1*, multiple DNA Polymerases, *spn-E*, *RnrL*, *RfC4*) (Buttitta et al., 2010; Dimova et al., 2003). However, we did not observe strong upregulation of other G1-S-promoting genes such as *dMyc* (1.52-fold change), *bantam*, *CycE* or *CycD*. When we compared gene expression signatures globally, we found a strong overlap (2.63-fold more genes than expected by chance) with a wounding and regeneration gene expression signature (Khan et al., 2017; Table S2). We also noted upregulation of several genes associated with proteotoxic and oxidative stress (*Xrp1*, multiple Glutathione S transferases, *AOX1* and specific DNA damage response genes) (Baumgartner et al., 2021). We found the strongest overlap of the *Nup98-96* knockdown signature with a cell competition ‘loser’ gene expression signature (5.67-fold more genes than expected by chance, 316/443 genes; Table S3), which is also known to activate chronic JNK signaling (Kucinski et al., 2017).

### *Nup98-96* knockdown leads to defects in protein synthesis

**Fig. 4.**
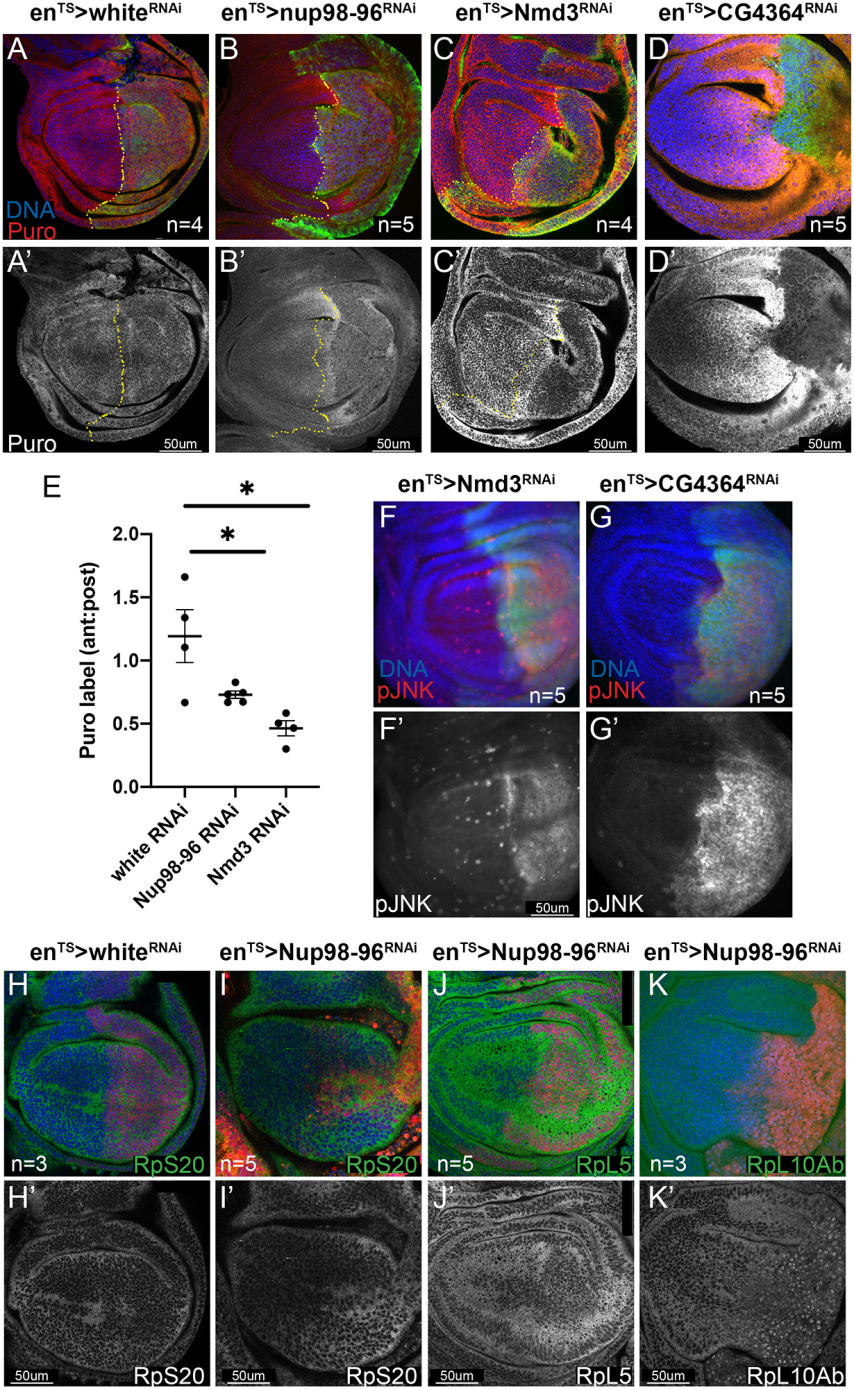
**Knockdown of Nup98-96 leads to ribosomal protein mislocalization and compromised protein synthesis.** (A-D′) Using *en^TS^*, the indicated *UAS*-RNAis were expressed in the posterior wing disc for 72 h prior to dissection of wandering L3 larvae and labeled for protein synthesis using O-propargyl-puromycin (puro) incorporation. Puro labeling experiments in discs were performed at multiple time points (10-20 min); data from one experiment with 12 min of labeling are shown. (E) The ratio of anterior:posterior puro-labeling is used to normalize for puro incorporation. *Nup98-96* and *Nmd3* knockdown reduced puro labeling (*P<0.05; unpaired Student's *t*-test). (F-G′) Knockdown of *Nmd3* or *CG4364* (Pescadillo homolog) for 48 h in the posterior wing disc using *en^TS^* activated JNK signaling. (H-K′) Using *enRFP*^TS^, the indicated *UAS*-RNAis were expressed for 72 h in backgrounds expressing GFP or YFP protein traps for the indicated Rp subunits. (K) RpL10Ab-YFP shows aberrant nuclear enrichment when Nup98-96 is knocked down. Plots of individual biological replicates include mean±s.e.m. Scale bars: 50 μm.

The strong overlap of the gene expression changes in *Nup98-96* knockdown with the cell competition ‘loser’ signature suggested to us that a proximal effect of Nup98 loss could be on ribosome biogenesis. We further examined a gene expression signature associated with Xrp1, an AT-Hook, bZip transcription factor that mediates signaling downstream of ribosomal protein mutations and proteotoxic stress (Langton et al., 2021; Lee et al., 2018). We found that a striking proportion of Xrp1 targets (115 of 159 overlapping in our dataset; Table S4) were upregulated when *Nup98-96* was knocked down (Ji et al., 2019). Consistent with a defect in ribosome function, we observed a decrease in protein synthesis when *Nup98-96* was knocked down in wings, as measured by a puromycin-labeling assay (Deliu et al., 2017) (Fig. 4A,B′). We did not observe downregulation of any ribosomal proteins in our RNAseq dataset, with the exception of a 2-fold decrease in *RpS19b*, which is a non-Minute, duplicated ribosomal protein gene with tissue-specific expression (Marygold et al., 2007). Any effects on *RpS19b* levels are likely buffered by its paralog *RpS19a*, which exhibits much stronger expression in larval wings and was unchanged by *Nup98-96* knockdown (Brown et al., 2014).

Nups play a key role in the nuclear export of ribosomal subunits in cooperation with the exportin chromosomal region maintenance 1 (CRM1; also known as Emb, exportin-1 or XPO1), which binds to nuclear export sequences to facilitate export of cargo proteins (Gleizes et al., 2001; Johnson et al., 2002; Moy and Silver, 2002; Oeffinger et al., 2004). We wondered whether the proximal defect in *Nup98-96* knockdown tissues might be defects in nuclear export of ribosomal complexes. First, we examined whether our partial knockdown of Nup98-96 function by RNAi was sufficient to disrupt nucleo-cytoplasmic localization, because previous work had suggested that knockdown of *Nup98-96* transcripts in *Drosophila* S2 cells did not produce such defects (Sabri et al., 2007). We confirmed that, by 52 h of knockdown with *en^TS^ in vivo*, we could easily visualize defects in nuclear localization of a ubiquitously expressed RFP with a nuclear localization signal (NLS), and, by 72 h of knockdown, nuclear localization of NLS-RFP was dramatically reduced (Fig. S4A). We next confirmed that knockdown of an essential component of the nuclear export machinery for ribosome subunits, *Nmd3* (Ma et al., 2017), also effectively reduced protein synthesis (Fig. 4C,C′). As a positive control, we also knocked down *CG4364*, the fly homolog of the pre-rRNA processing component Pescadillo (Lapik et al., 2004) (Fig. 4D-E). Inhibition of ribosome export machinery and pre-rRNA processing were both sufficient to induce strong phosphorylation of JNK (Fig. 4F-G′) in the wing disc.

Ribosome large and small complexes are exported from the nucleus separately as assembled pre-ribosomal particles and must associate with cytoplasmic maturation factors to exchange specific components to form mature functional ribosomes (Lo et al., 2010). We screened through collections of endogenously tagged Rp subunits and found that RpL10Ab, but not other Rp subunits (RpS20 and RpL5), were mislocalized when *Nup98-96* was knocked down (Fig. 4H-K′). Interestingly, the defect in RpL10Ab localization was nuclear retention, the opposite of the effect of *Nup98-96* knockdown on NLS-RFP. RpL10Ab (also called L10a or uL1) is required to associate with Nmd3 for efficient pre-60S nuclear export (Musalgaonkar et al., 2019). Normally, RpL10Ab is translated in cytoplasm, localized to the nucleolus for assembly into the pre-60S complex and then exported bound to the Nmd3 adaptor. The nuclear retention of RpL10Ab upon *Nup98-96* knockdown was initially puzzling as the other RpL subunits examined did not exhibit similar localization defects. However, recent work has revealed that, in mammals, RpL10A is associated with a subset of specialized ribosomes and is not found in all 60S complexes (Shi et al., 2017). We suggest that knockdown of *Nup98-96* partially compromises protein synthesis by inhibiting proper cytoplasmic translocation of a subset of pre-60S subunits that are RpL10Ab associated. Importantly, *RpL10Ab* is not a Minute gene (Marygold et al., 2007), possibly because it is a sub-stoichiometric ribosome component. Consistent with this, we do not recover significant overlap with the proteasomal stress portion of the ‘loser’ gene expression signature when Nup98-96 is compromised (Baumgartner et al., 2021), again suggesting that protein synthesis is only partially reduced when Nup98-96 function is compromised.

### *NUP98-96* knockdown in human cells leads to defects in protein synthesis and JNK activation

**Fig. 5.**
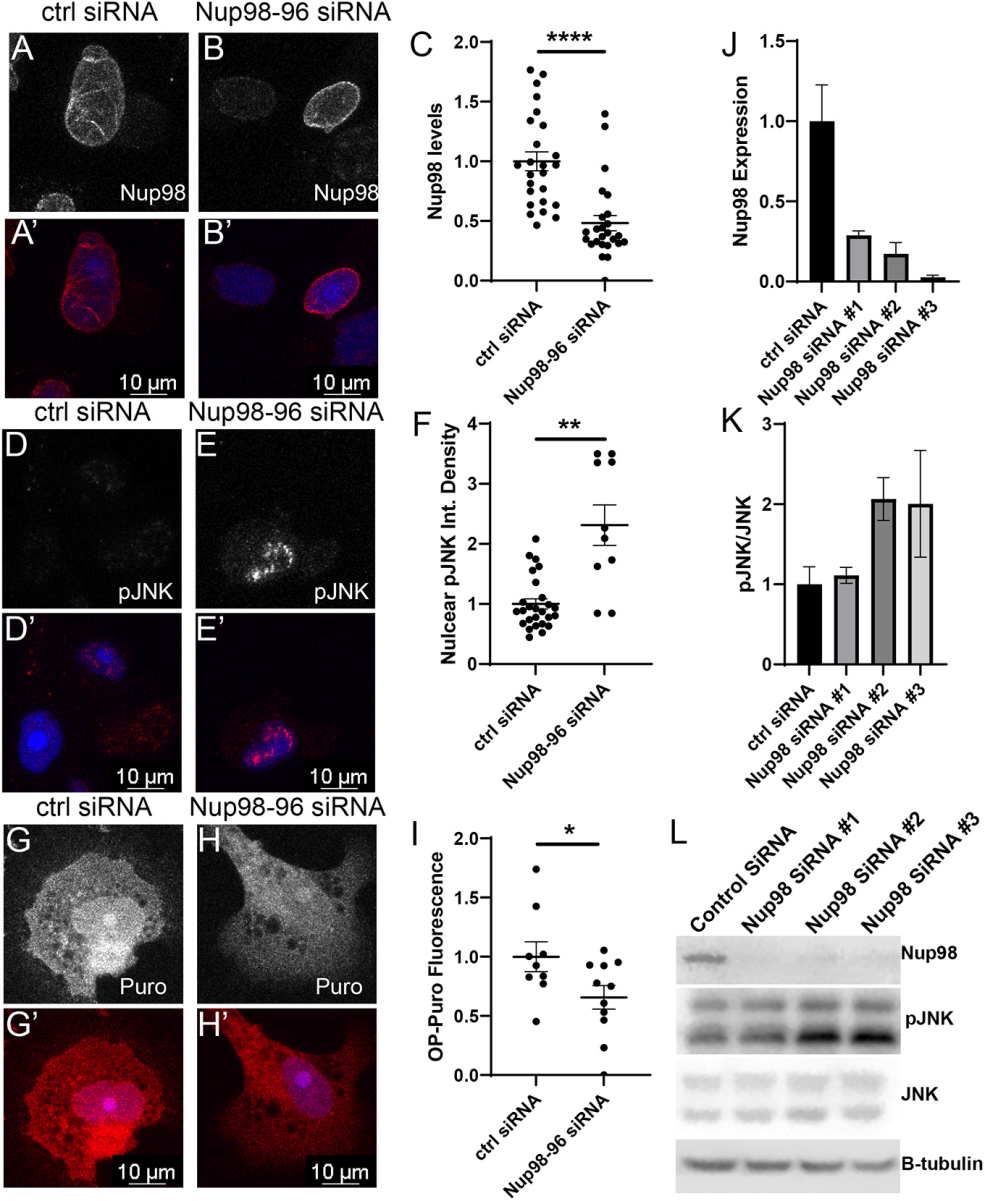
**Knockdown of Nup98-96 in human cells leads to reduced protein synthesis and JNK signaling.** (A-B′,D-E′,G-H′) PC3 cells were treated with small interfering (si)RNAs for 72 h, and cells were either fixed and stained with anti-Nup98 antibody (A-B′) or pJNK (D-E′), or labeled with puro for 12 min (G-H′). Control siRNA (ctrl) is a scrambled siRNA. (C,F,I) *NUP98* siRNA reduces Nup98 levels (C) as well as reduces protein synthesis (F) and increases pJNK labeling (I). (J-L) Western blot analysis of PC3 cells treated with Ctrl and *NUP98* siRNAs (L) shows that *NUP98* siRNAs reduced the protein level of Nup98 (J) as well as increased phosphorylated JNK (K). Quantifications of fluorescence were performed on individual cells from three replicates from at least two independent experiments. Plots of individual biological replicates include mean±s.e.m. Quantifications for the western blots were done in triplicate for three different sets of siRNAs (*P<0.05, **P<0.01, ****P<0.0001; unpaired Student's *t*-tests; F uses Welch's correction for unequal sample size). Scale bars: 10 μm.

As described in the Introduction, there is abundant evidence that loss of Nup98-96 function might contribute to tumorigenesis. We wondered whether inhibition of Nup98-96 in mammalian cells would also impact protein synthesis and JNK signaling as we observe in *Drosophila*. Of note, a screen for factors involved in ribosome biogenesis in HeLa cells identified several Nups containing FG repeats, including Nup98 as hits involved in pre-60S export, suggesting that Nup98 effects on protein synthesis will be broadly conserved (Wild et al., 2010). We used siRNA to *NUP98-96* in MCF7 breast cancer cells and PC3 prostate cancer cells for 72 h and compared effects on Nup98 protein levels, protein synthesis and pJNK to a control scrambled siRNA. We found that siRNA to *NUP98-96* was sufficient to reduce protein synthesis and increase phosphorylation of JNK in both cell types (Fig. 5A-H′; Fig. S5).

### Overexpression of Nup98 leads to defects in protein synthesis and JNK activation

**Fig. 6.**
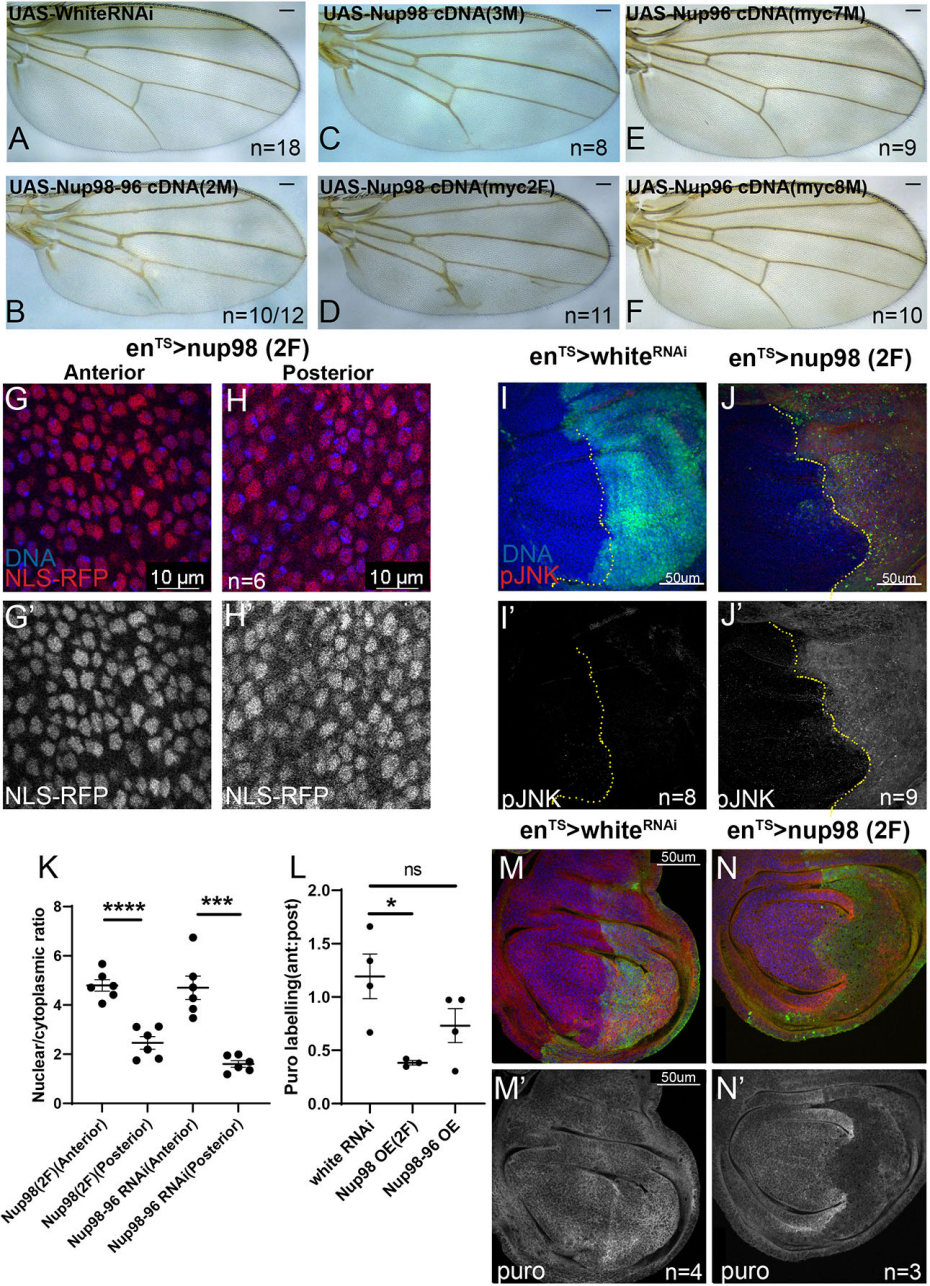
**Overexpression of Nup98 disrupts protein synthesis and activates JNK signaling.** (A-F) Using *en^TS^*, the indicated *UAS*-cDNA constructs were expressed in the posterior wing from mid-L2 and adult wings were mounted. Overexpression of Nup96 had no effect on the posterior wing, while overexpression of Nup98 or Nup98-96 reduced posterior wing size and disrupted vein patterning. Scale bars: 100 μm. (G-H′) Using *en^TS^*, a ubiquitous RFP-NLS was expressed with *UAS-Nup98* 2F for 24 h. The nuclear:cytoplasmic ratio for RFP-NLS was quantified and shown for the anterior wing disc (no Nup98 expression) and posterior wing disc (Nup98 overexpression). Ratios are also provided for *Nup98-96* RNAi (from Fig. S4) for comparison. Scale bars: 10 μm. (I-J′) Using *en*^*TS*^, *Nup98-96* cDNA was expressed in the posterior wing disc for 72 h prior to dissection of wandering L3 larvae and labeling with pJNK. UAS-*white* RNAi serves as a negative control, showing that endogenous pJNK at this stage is very low. Scale bars: 50 μm. (K-N′) Using *en^TS^*, the indicated *UAS*-cDNA or RNAi was expressed for 72 h prior to dissection and labeling with puro to measure protein synthesis. Overexpression of Nup98 2F reduced protein synthesis in the posterior disc, while Nup98-96 overexpression had a milder effect (ns, not significant; *P<0.05, ***P<0.005, ****P<0.0001; unpaired Student's *t*-test). Plots of individual biological replicates include mean±s.e.m. Scale bars: 50 μm

Most of the attention on Nup98 translocations in cancer has focused on overexpressing Nup98 fusion partners. However, when overexpressed, Nup98 has been shown to behave as dominant negative and to disrupt the nuclear envelope and nuclear transport (Fahrenkrog et al., 2016; Mendes et al., 2020), possibly by forming phase-separated aggregates outside the nuclear pore (Ahn et al., 2021; Schmidt and Gorlich, 2015). We noted that Nup98 overexpression in the posterior wing disc reduced tissue size and, in severe cases, disrupted pattering (Fig. 6A-F). We therefore examined whether Nup98 overexpression in the *Drosophila* wing disc mimicked aspects of Nup98-96 inhibition, as described for other *Drosophila* tissues (Pascual-Garcia et al., 2014). Overexpression of a strong *UAS-Nup98* cDNA construct (2F) reduced nuclear localization of an NLS-tagged RFP, resulting in increased cytoplasmic accumulation and a reduced nuclear:cytoplasmic ratio (Fig. 6G-K). Overexpression of a *UAS-Nup98* cDNA construct was also sufficient to increase cell death and activate JNK signaling in the posterior wing disc (Fig. 6I-J′), and overexpression of both *UAS-Nup98* and *UAS-Nup96* or *UAS-Nup98* alone (2F) reduced protein synthesis levels (Fig. 6L-N′). We suggest that Nup98-96 acts as a ‘goldilocks’ gene (Braune and Lendahl, 2016), where too much or too little activity leads to chronic stress signaling and increased cellular turnover, potential hallmarks of tumorigenesis. This complication might explain why this locus is particularly prone to misregulation by translocations in cancer, which would reduce Nup98-96 normal functions and simultaneously provide additional Nup98-containing fusion proteins.

## DISCUSSION

### Partial Nup98-96 loss of function leads to paradoxical increases in cell cycling and cell death accompanied by reduced protein synthesis

Protein synthesis and the cell cycle are usually coupled by pathways such as insulin and TOR signaling as well as growth and cell cycle checkpoints, which promote or limit cell cycle progression and protein synthesis coordinately (Grewal, 2009; Lockhead et al., 2020; Romero-Pozuelo et al., 2017, 2020). Here, we describe a seemingly paradoxical situation in which protein synthesis and the cell cycle are effectively uncoupled. When Nup98 and Nup96 are partially compromised, cells with reduced protein synthesis cycle more and even bypass developmentally induced G1 arrests. This is accompanied by high levels of chronic JNK signaling and induction of apoptosis, along with expression of genes involved in tissue regeneration and compensatory proliferation. When apoptosis is blocked using the caspase inhibitor P35, tissue overgrowth and mispatterning results, reminiscent of tumorigenesis. We propose that mutations or gene expression changes that reduce Nup98 and Nup96 function, in the presence of apoptosis suppression, can contribute to tumorigenesis. This may help to explain contexts of Nup98 and/or Nup96 loss that could predispose for cancer (Franks and Hetzer, 2013; Simon and Rout, 2014; Singer et al., 2012).

The phenotype we describe here for *Nup98-96* inhibition is strikingly similar to that recently described for a ribosomal protein mutant, when cell death is blocked (Akai et al., 2021). When we examined the gene expression signature in response to reduced *Nup98-96*, we observed a strong overlap with conditions of reduced protein synthesis caused by stoichiometric imbalances in ribosomal proteins (Kucinski et al., 2017; Lee et al., 2018). We suggest that this effect of *Nup98-96* inhibition is due to defects in nucleo-cytoplasmic transport of RpL10A, although we cannot rule out that localization of other ribosomal proteins may also be affected. Because the defect is in RpL10A localization, rather than levels, we were unable to rescue the *Nup98-96* knockdown phenotypes with RpL10A overexpression. On the contrary, we observed several stress signaling phenotypes when we overexpressed RpL10A itself even in a wild-type background, suggesting that RpL10A levels must also be carefully controlled (Chaichanit et al., 2018; Wonglapsuwan et al., 2011). This may be of broader consequence to the *Drosophila* research community because Gal4/*UAS*-driven overexpression of this ribosomal protein is used for translatome profiling through translating ribosome affinity purification (Thomas et al., 2012). Importantly, localization of 40S and 60S subunits is not globally disrupted in our *Nup98-96* knockdown conditions, and protein synthesis is only partially reduced. We suggest that this is because RpL10A is a sub-stoichiometric component of ribosomes and that only the subset of ribosomes containing RpL10A are affected. In mammals, RpL10A-containing ribosomes have been shown to translate genes required for cell survival and are depleted of those required for cell death (Shi et al., 2017). Whether this is the case for *Drosophila* RpL10A-containing ribosomes remains to be determined, although increasing RpL10A expression in *Drosophila* has been shown to affect E-cadherin and InR levels, suggesting that components of these pathways could be regulated by RpL10A levels (Chaichanit et al., 2018).

The effects of reducing *Nup98-96* expression are likely to be pleiotropic, and we cannot rule out the possibility that Nup98 and Nup96 misregulation may also lead to more direct effects on the cell cycle, independent of JNK signaling and reduced protein synthesis. Indeed, when JNK signaling is blocked by a dominant negative, overall compensatory proliferation is significantly reduced, but Nup98-96-reduced tissue still exhibits a slightly higher mitotic index than tissue with normal Nup98-96 levels. This could be, in part, the result of a known Nup98 interaction with the anaphase-promoting complex/cyclosome (APC/C) which leads to aneuploidy when Nup98 levels are reduced (Jeganathan et al., 2006, 2005). This interaction with the APC/C may also explain the disruption of terminal cell cycle arrest caused by reduced Nup98-96, as high APC/C activity promotes proper timing of the final cell cycle (Buttitta et al., 2010; Reber et al., 2006; Ruggiero et al., 2012; Tanaka-Matakatsu et al., 2007). We tested for aneuploidy using flow cytometry on wing discs and did not observe obvious accumulation of aneuploidy when *Nup98-96* is knocked down, either with or without apoptosis inhibition. Alternatively, effects on nuclear export of cell cycle factors or their mRNAs may also contribute to the cell cycle phenotypes (Chakraborty et al., 2008), although we did not find obvious changes in protein levels or dynamics of Cyclins A or B. We also examined whether misregulation of transcriptional targets of Nup98 regulated through off-pore roles may explain the phenotypes we observe, but we did not find significant overlap of genes altered in our *Nup98-96* knockdown with Nup98-bound targets determined by chromatin immunoprecipitation with sequencing (ChIP-seq) in larval brains (Pascual-Garcia et al., 2017) or Nup98-regulated genes identified by RNAseq in S2 cells (Kalverda et al., 2010). We found a mild enrichment (1.43-fold over that expected by chance) in the overlap of genes altered in our *Nup98-96* knockdown with Nup98 ChIP-seq targets in S2 cells (Pascual-Garcia et al., 2017; Table S5). Overall, the previously described wounding/regeneration and ‘loser’ gene expression programs explain nearly half (49.7%) of the gene expression changes we observe in wing discs when Nup98-96 is reduced (Fig. 3), suggesting that these may be the main drivers of the phenotypes we observe.

### Potential for AIP in Nup98 cancers

Blocking apoptosis in cells with inhibited *Nup98-96* leads to phenotypes consistent with sustained AIP, which is thought to contribute to tumorigenesis in epithelia (Fogarty and Bergmann, 2017). Epithelial tumors exhibit wounding phenotypes, chronic inflammation and cell death (Dvorak, 1986; Karin and Clevers, 2016). Chronic AIP leads to sustained proliferation and results in abnormal, hyperplastic overgrowth (Perez-Garijo et al., 2009; Pinal et al., 2018). AIP, therefore, could contribute to overproliferation in epithelial cancers with disrupted *Nup98-96* expression (Perez-Garijo, 2018). AIP has been suggested to occur in colorectal cancer and melanoma (Bordonaro et al., 2014; Donato et al., 2014), both of which have been suggested to exhibit Nucleoporin misregulation (Roy and Narayan, 2019). How this might relate to aberrant signaling in hematological malignancies related to Nup98 misexpression is unclear. It is possible that the effects of Nup98 misregulation impact different tissue types through similar pathways that impinge on distinct downstream target genes in different tissues. For example, expression of a NUP98-HOXA9 fusion in a *Drosophila* model with a normal *Nup98-96* locus leads to hyperplastic overproliferation in hematopoietic tissues but minimal effects in epithelial tissues (Baril et al., 2017), while loss of *Nup98-96* in larval hematopoietic tissues leads to a loss of progenitors, a phenotype also observed upon inhibition of the ribosomal protein RpS8 (Mondal et al., 2014). *NUP98* mutations in leukemias are associated with mutations affecting apoptosis, such as *BCR-ABL*, *NRAS*, or *KRAS* and *ICSBP* (also known as *IRF8*) (Gabriele et al., 1999; Gough et al., 2011; Gurevich et al., 2006; Hu et al., 2016; Slape et al., 2008). Mouse models with Nup98 protein fusions exhibit increased apoptosis (Choi et al., 2008; Lin et al., 2005), and a zebrafish model of NUP98-HOXA9-driven leukemia upregulates Bcl2 to suppress apoptosis (Forrester et al., 2011). In a mouse model of Nup98-HoxD13-driven leukemia, loss of p300 leads to reduced apoptosis and enhanced activation of JAK/STAT signaling, reminiscent of signaling effects we see in AIP (Cheng et al., 2017). In our *Nup98-96* RNAi experiments, we reduced Nup98 protein levels to ∼50-70% of the normal level, consistent with other studies using this RNAi approach (Pascual-Garcia et al., 2014). Our data suggest that this locus can behave as a dominant negative when the Nup98 portion is overexpressed through translocations as well as a haplo-insufficient tumor suppressor in some contexts. We propose that disruption of the *NUP98-96* locus in cancers with or without *NUP98* translocations may contribute to tumorigenesis through aberrant JNK signaling and AIP, in the presence of additional hits that block cell death.

## MATERIALS AND METHODS

### Fly stocks

Fly stocks used are listed in the Supplementary Materials and Methods.

### Immunofluorescence

*Drosophila* samples were fixed in 4% paraformaldehyde/1× PBS solution for 20-30 min, rinsed twice in 1× PBS with 0.1% Triton X-100 detergent (1× PBST). The samples were then incubated in an appropriate dilution of antibodies in PAT [1× PBS+0.1% Triton X-100+1% bovine serum albumin (BSA)] for 4 h at room temperature or overnight at 4°C. The samples were then washed three times for 10 min in 1× PBST and incubated in secondary antibody conjugated with required fluorophore for 4 h in PBT-X+2% normal goat serum (1× PBS+0.3% Triton X-100+0.1% BSA) at room temperature or overnight at 4°C. As a nuclear counterstain, 4′,6-diamidino-2-phenylindole (DAPI) or Hoechst 33258 was used, and samples were mounted on glass slides using 5 µl Vectashield mounting medium (Vector Laboratories). Slides were imaged using a Leica DMI6000 epifluorescence system with subsequent deconvolution or a Leica SP5 confocal microscope.

For PC3 and MCF-7 cells, fixation and washes were performed as described above, except in 12-well dishes or eight-chamber slides, with just 1 h of incubation with primary and secondary antibodies at room temperature. Experiments for each siRNA were performed in triplicate.

Sample sizes are indicted on figures, and penetrance, when not 100%, is indicated as the fraction of individuals showing the phenotype (numerator)/total sample size (denominator). For adult *Drosophila* wings, we mounted only one wing per individual; therefore, the sample number represents biological replicates. For larval experiments, we did not keep track of biological versus technical replicates (e.g. two wings per individual); therefore, *n*-values represent both biological and technical replicates (a maximum of two) processed together. Crosses for several of the experiments were repeated multiple times, or at different time points or with multiple independent RNAi lines, as indicated in the text.

### EdU labeling and pulse-chase assay

Crosses were flipped every day and kept at room temperature (22°C). For EdU labeling in Fig. 1K-L′ (labeling post-dissection), larvae were dissected inverted and incubated in 10 µM EdU prior to fixation and labeling. The post-dissection EdU labeling was performed three independent times with EdU labeling intervals of 2, 5 and 10 min. Data from the 5 min labeling are shown. For the EdU pulse-chase assay, vials with embryos were transferred to 29°C after 2 days. Larvae at mid-L3 (∼66 h after the transfer) were removed from the vials by floating in 30% sucrose/1× PBS solution. The larvae were transferred to a vial with YG food mixed with 100 µM EdU and blue food coloring (to track feeding) at 29°C for 1 h. Larvae with blue abdomens were then transferred to fresh non-EdU food (chase) for 6-8 h at 29°C (equivalent to 7-9 h at 25°C). EdU pulsed-chased wandering L3 larvae were collected, dissected, fixed, and antibody stained for EdU, PH3 and GFP (to mark the A-P compartment boundary). The EdU labeling was performed using a Click it EdU-555 kit (C10338, Invitrogen) following the manufacturer's instructions. The slide was then imaged using confocal microscopy, and the total number of cells positive for both EdU and PH3 were scored and normalized to the total mitotic index. This experiment was replicated three independent times for 6, 7 and 9 h pulse-chase intervals, with at least five animals per replicate. Data for the 7 h replicate are shown.

### Protein synthesis puromycin assay

L3 larvae were dissected in Ringer's solution (Sullivan et al., 2000), and inverted larvae heads containing wing discs were incubated with 20 µm O-propargyl-puromycin (OPP; Invitrogen) in Ringer's solution for 12 min. The sample was then fixed with 4% paraformaldehyde/1× PBS solution for 20 min, and labelled using the Click-it OPP kit (C10457, Invitrogen), following the manufacturer's instructions.

### Antibodies

Antibodies used are listed in the Supplementary Materials and Methods.

### siRNA in mammalian cells

MCF7 cells were a gift from S. Merajver’s laboratory (University of Michigan). PC3 cells were a stable cell line expressing cell cycle reporters hCdt1-mCherry and p27K-mVenus previously described (Takahashi et al., 2019). The cells were grown to 50-70% confluency in a 12-well plate or eight-well chamber slide. The cells were then transfected with 20 nM *Nup98* siRNA or control siRNA using Lipofectamine RNAi MAX (Invitrogen), following the manufacturer's protocol. The cells were incubated with the indicated siRNA for 72 h, then harvested for fixation and staining or lysed for western blotting. siRNAs used were as follows: Silencer Select Negative Control No. 1 (4390843, ThermoFisher Scientific); *Nup98-96* siRNA#1, Silencer Pre-designed siRNA (AM16708, ThermoFisher Scientific); *Nup98-96* siRNA#2, Silencer Select Pre-designed siRNA (4392420, ThermoFisher Scientific); *Nup98-96* siRNA#3, *Nup98* siRNA (sc-43436, Santa Cruz Biotechnology). Cell lines were tested for mycoplasma routinely and were negative in June 2021. PC3 cells were authenticated prior to publication (Takahashi et al., 2019).

### Image analysis and quantification

Image quantification was performed using FIJI. For quantification of DCP1, PH3 or pJNK labeling in Figs 1 and 2, regions of similar size (ROIs) in the anterior and posterior wing disc were hand-drawn using the nuclear (DAPI or Hoechst 33258) staining to indicate tissue boundaries and GFP labeling for compartment boundaries. Integrated density of labeling was normalized to ROI area for *white* RNAi and *Nup98-96* RNAi under conditions blinded to sample identity. Area-normalized integrated density with subtraction of background ROIs outside of the tissue was used for EdU, PH3, Nup98 and puromycin quantification. For ratios in the EdU/PH3 pulse-chase assay, double-labeled cells were counted in each compartment, and the ratio normalized to total mitotic index across wing discs is shown. Each dot in the scatter plot represents an individual wing disc from a different animal (for Figs 1, 2 and 4) or individual cells from experiments performed in triplicate (Fig. 5).

### Mounting and imaging of adult wings

Adult wings were preserved in ethanol, washed in methyl salicylate and mounted in Canada Balsam (Sigma-Aldrich) as described (O'Keefe et al., 2012). Adult wings were photographed under brightfield conditions on a Leitz Orthoplan2 at 5× magnification, using a Nikon DS-Vi1 color camera and Nikon NIS Elements software.

### RNAseq

Experimental animals were of the genotype UAS-P35/w; *ap*-Gal4, UAS-GFP/+; *tub*-gal80^TS^/UAS-*Nup98-96* RNAi TRiP. Control animals were of the genotype UAS-P35/w; *ap*-Gal4, UAS-GFP/+; *tub*-gal80^TS^/UAS-*white* RNAi TRiP. Crosses were performed at room temperature, and embryos were collected within a 12 h window to synchronize developmental staging and shifted to 18°C. Animals were reared in uncrowded conditions (70 larvae per vial). On day 4, animals were transferred to 28°C, and, 72 h later, third instar wing discs were dissected in sterile 1× PBS. We followed a Trizol-based RNA preparation protocol with dounce homogenization of 40 wing discs per sample with three replicated per genotype, as previously described (Flegel et al., 2016).

Using PolyA selection, the University of Michigan's Sequencing Core generated barcoded libraries for each sample and confirmed the quality via the Bioanalyzer and qPCR. Sequencing was performed with the Illumina HiSeq 2000 platform and high-read quality was confirmed using FastQC. Reads were aligned to the BDGP6.82 *D. melanogaster* genome using Rsubread (v1.21.5), with featureCounts resulting in >77% of the reads being successfully assigned to genes (Liao et al., 2014). Counts per million (cpm) were determined with edgeR (v3.13.4), and transcripts with low expression were identified and removed using the data-based Jaccard similarity index determined with HTSFilter (v1.11.0). The cpm were TMM normalized (calcNormFactors), voom transformed (Law et al., 2014) and fitted to a linear model (lmFit), then differential gene expression calls were made with eBayes. The full dataset is available at Gene Expression Omnibus (GEO) (GSE152679). Differentially expressed genes were defined as having a log_2_ fold change of ±0.5 (1.42-fold change) and adjusted *P*-value <0.05 (Table S1). For significance of overlap in differentially expressed genes with other datasets (Fig. 3), hypergeometric probabilities were calculated using the hypergeometric distribution as described (Flegel et al., 2016). For significance of overlap with previously published Nup98 ChIP-seq, our list of differentially expressed genes was compared to lists of genes near Nup98 ChIP-seq peaks and examined for overlap greater than that expected by chance using the hypergeometric distribution.

## Supplementary Material

Supplementary information
