# Supplementary material for: Misregulation of Nucleoporins 98 and 96 leads to defects in protein synthesis that promote hallmarks of tumorigenesis
Source: Dis Model Mech. 2022 Mar 16;15(3):dmm049234. doi: 10.1242/dmm.049234 (PMC8938402; doi:10.1242/dmm.049234)
Supplement: Supplementary information [file dmm-15-049234-s1.pdf]

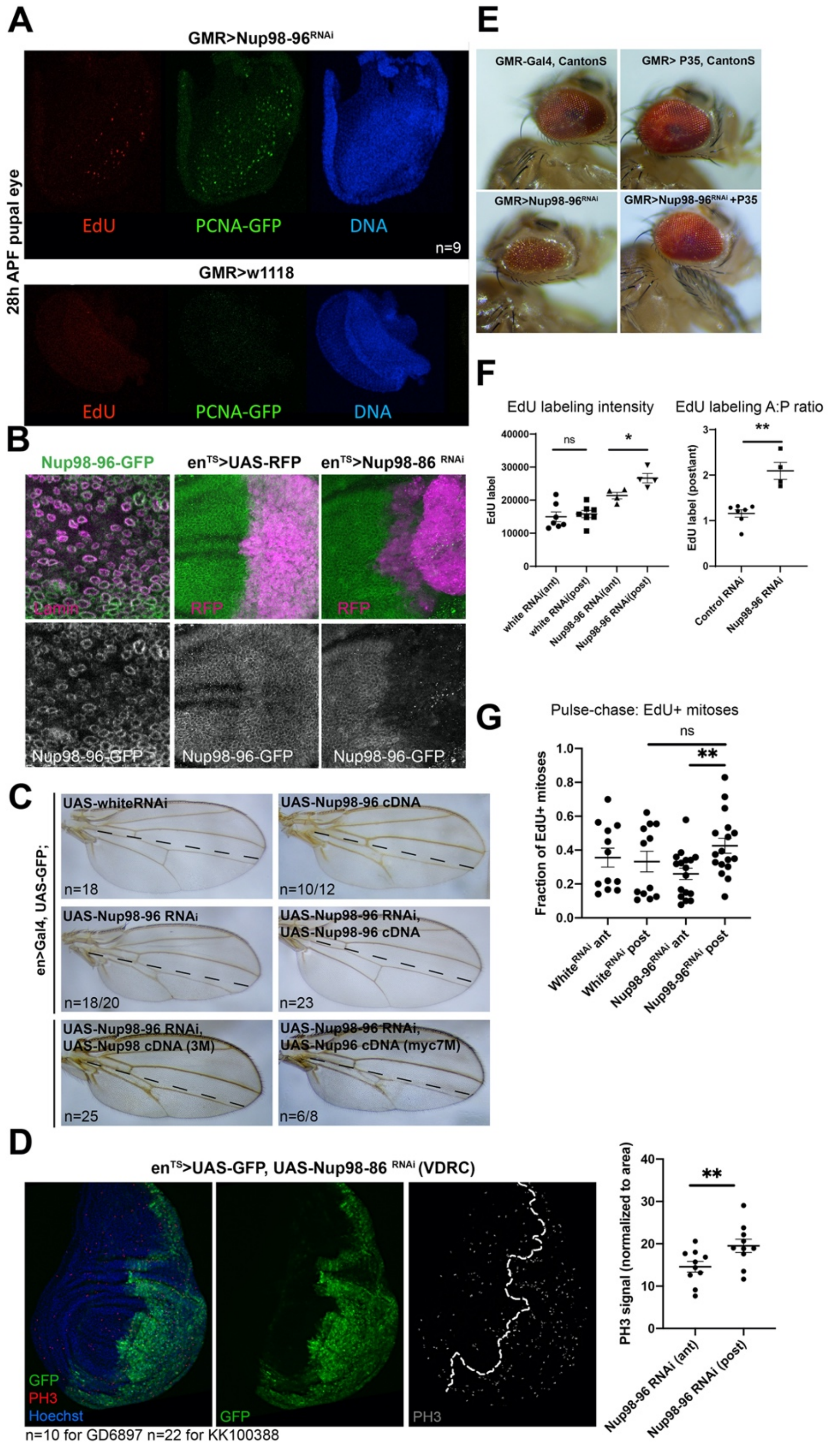

Disease Models & Mechanisms • Supplementary information

Fig S1.

(A) 28h APF pupal eyes were labeled for 2h with EdU to detect S-phase and stained for GFP to detect *PCNA* promoter driven GFP. S-phases and *PCNA* promoter activity is evident when RNAi to *Nup98-96* is driven with *GMR*-Gal4. *GMR*-Gal4 without any RNAi (in a w^{1118} background) serves as a control. Genotypes are: (top) w; *GMR*-Gal4 /+; *UAS-Nup98-96 RNAi* (TRiP)/*PCNA* prom-GFP (bottom) w; *GMR*-Gal4/+; *PCNA* prom-GFP/+.

(B) *Nup98-96-GFP* is from the FlyFos collection containing a Fosmid on II with *Nup98-96* coding region plus regulatory DNA and a GFP tag (Sarov et al., 2016) We confirmed this line exhibits the expected ubiquitous nuclear envelop labeling by co-staining with Lamin Dm0. (Right) We co-expressed Nup98-96-GFP in a background with *en-Gal4* driving RFP alone or RFP in combination with *UAS-Nup98-96 RNAi* (TRiP), we confirmed effective knockdown of Nup98-96-GFP in the *en-Gal4* expressing domain. Genotypes are: (left) *w; Nup98-96-GFP;* + (middle) *w; Nup98-96-GFP/ enGal4, UAS-RFP*; + (right) *w; Nup98-96-GFP/ enGal4, UAS-RFP; UAS-Nup98-96 RNAi (TRiP)/+.*

(C) cDNA rescue constructs providing *UAS-Nup98*, *UAS-Nup96* or *UAS-Nup98-96* were tested for the ability to rescue the wing phenotypes caused by *Nup98-96 RNAi*. Only expression of both *Nup98* and *Nup96* (middle right) fully rescued posterior wing size. Note that over-expression of both *Nup98* and *Nup96* without RNAi also led to reduced posterior wing size (see Fig. 6). Genotypes are: (top left) *w; en-Gal4, UAS-GFP/+; UAS-white RNAi (TRiP)/+* (top right) *w; en-Gal4, UAS-GFP/+; UAS-Nup98-96* (middle left) *w; en-Gal4, UAS-GFP/UAS-Nup98-96 RNAi* (VDRC GD); *UAS-white RNAi* (TRiP)/+ (middle right) *w; en-Gal4, UAS-GFP/UAS-Nup98-96 RNAi* (VDRC GD); *UASNup98-96 cDNA*/+ (bottom left) *w; en-Gal4, UAS-GFP/UAS-Nup98-96 RNAi* (VDRC GD); *UAS-Nup98 cDNA 3M*/+ (bottom right) *w; en-Gal4, UAS-GFP/UAS-Nup98-96 RNAi* (VDRC GD); *UAS-Nup96 cDNA 7M*/+.

(D) Two independent RNAi lines from the VDRC gave similar phenotypes to the TRiP RNAi in the larval wing disc. Line GD6897 is shown at wandering L3 after 72h of expression at 28°C with PH3 labeling for mitoses, similar results were obtained with the line KK100388. Genotype: *w; en-Gal4, UAS-GFP; UAS-Nup98-96 RNAi GD6897; Tub-Gal80*^{TS}/ +. Quantifications of PH3 for 10 wings of the genotype *w; en-Gal4, UAS-GFP; UAS-Nup98-96 RNAi KK100388; Tub-Gal80*^{TS}/ + are shown at right. PH3 signal is significantly increased in the posterior domain. **P<0.01 by a two-tailed paired t-test. Plots of individual biological replicates include mean±s.e.m.

(E) Controls related to Fig 1 E-F. *GMR*-Gal4 driving *Nup98-96 RNAi* alone results in a smaller, rough eye, likely due to increased apoptosis, which can be partially rescued by co-expressing *GMR>P35*.

(F) Quantifications of EdU labeling related to Fig. 1 K-L. *Nup98-96* knockdown results in increased EdU labeling in the posterior wing disc compared to the anterior and compared to *white* RNAi controls. Experiments were repeated independently for 2min, 5 min, and 10 min. of labeling. Genotypes: *w; en-Gal4, UAS-GFP/ + ; Tub-Gal80*^{TS}/ *UAS-white RNAi* or *w; en-Gal4, UAS-GFP/ + ; Tub-Gal80*^{TS}/ *UAS-Nup98-96 RNAi*. Plots of individual biological replicates include mean±s.e.m.

(G) Related to EdU pulse-chase experiment in Fig. 1M-O. Quantification of EdU+ mitoses in wing discs for 7h EdU pulse-chase experiment. *Nup98-96* knockdown results in an increased fraction of cells progressing from S-M in 7h in the posterior wing disc (autonomous effect) compared to the anterior (non-autonomous effect), but the increase

Disease Models & Mechanisms • Supplementary information

with *Nup98-96* knockdown is only ~20% and not statistically significant when compared to an external *white RNAi* control (mean for *Nup98-96 RNAi* = 0.425, mean for *white RNAi* = 0.332). *Nup98-96 RNAi* anterior to posterior comparison, **P<0.01 by two-tailed paired t-test. *white RNAi* posterior to *Nup98-96 RNAi* posterior comparison not significant (n.s.) by unpaired t-test with Welch's correction for unequal sample size. Plots of individual biological replicates include mean±s.e.m. Genotypes: *w; en-Gal4, UAS-GFP/ + ; Tub-Gal80*^{TS}*/ UAS-white RNAi* or *w; en-Gal4, UAS-GFP/ + ; Tub-Gal80*^{TS}*/ UAS-Nup98-96 RNAi*.

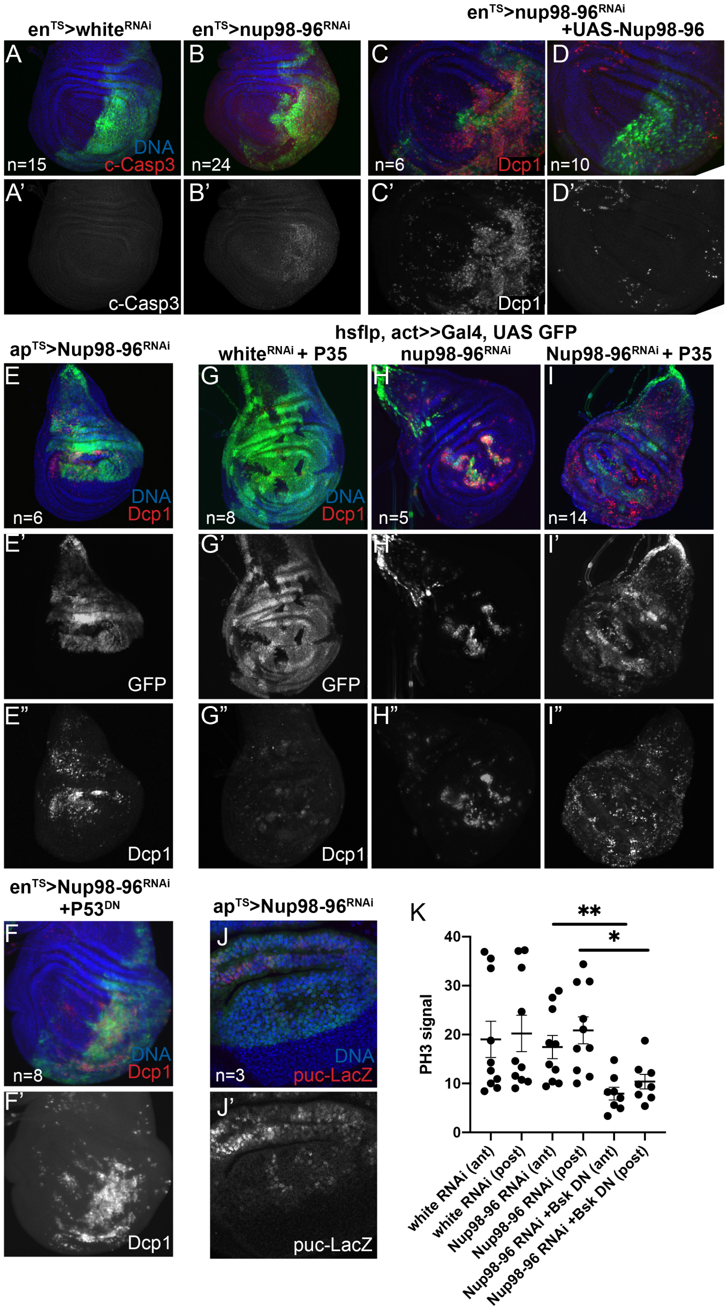

Disease Models & Mechanisms • Supplementary information

Fig S2. Nup98-96 knockdown leads to apoptosis which is rescued by co-expression of UAS-Nup98-96 cDNA. The indicated transgenes were driven by *en-Gal4*, with *UAS GFP* for 72h prior to dissection using *tub-Gal80^{TS}*. (A,B) Cleaved caspase 3 labeling indicates apoptosis in the posterior compartment when Nup98-96 is knocked down. (C,D) Co-expression of *Nup98-96* cDNA rescues the apoptosis caused by Nup98-96 knock-down, as assessed with Dcp-1. (E) Expression of *Nup98-96 RNAi* in the dorsal compartment (using *ap-Gal4*, *UAS-GFP*; *tub-Gal80^{TS}*) also leads to apoptosis. (F) Co-expression of p53 dominant negative with *Nup98-96 RNAi* in the posterior compartment (using *en-Gal4*, *UAS-GFP*; *tub-Gal80^{TS}*) does not rescue apoptosis. (G-I) Expression of *Nup98-96 RNAi* in clones throughout the wing pouch (using *hs-flp with act>stop>-Gal4, UAS-GFP*) also leads to apoptosis within clones, while co-expression with P35 (using *hs-flp with act>stop>-Gal4, UAS-GFP, UAS-P35*) suppresses apoptosis within clones and leads to apoptosis outside of clones expressing *Nup98-96 RNAi.* (J) Expression of *Nup98-96 RNAi* in the dorsal compartment (using *ap-Gal4*, *UAS-GFP*; *tub-Gal80^{TS}*) leads to upregulation of puc-LacZ expression (from *puc^{e69}* allele), a hallmark of JNK signaling. (K) Quantifications of PH3 signal broken down by ant/post compartment. *Nup98-96 RNAi* and *Bsk DN* are expressed only in the posterior using *en-Gal4*, *UAS-GFP*; *tub-Gal80^{TS}*. Note that overall PH3 labeling is reduced in both compartments when JNK signaling is inhibited with *Bsk DN*. Plots of individual biological replicates include mean±s.e.m.

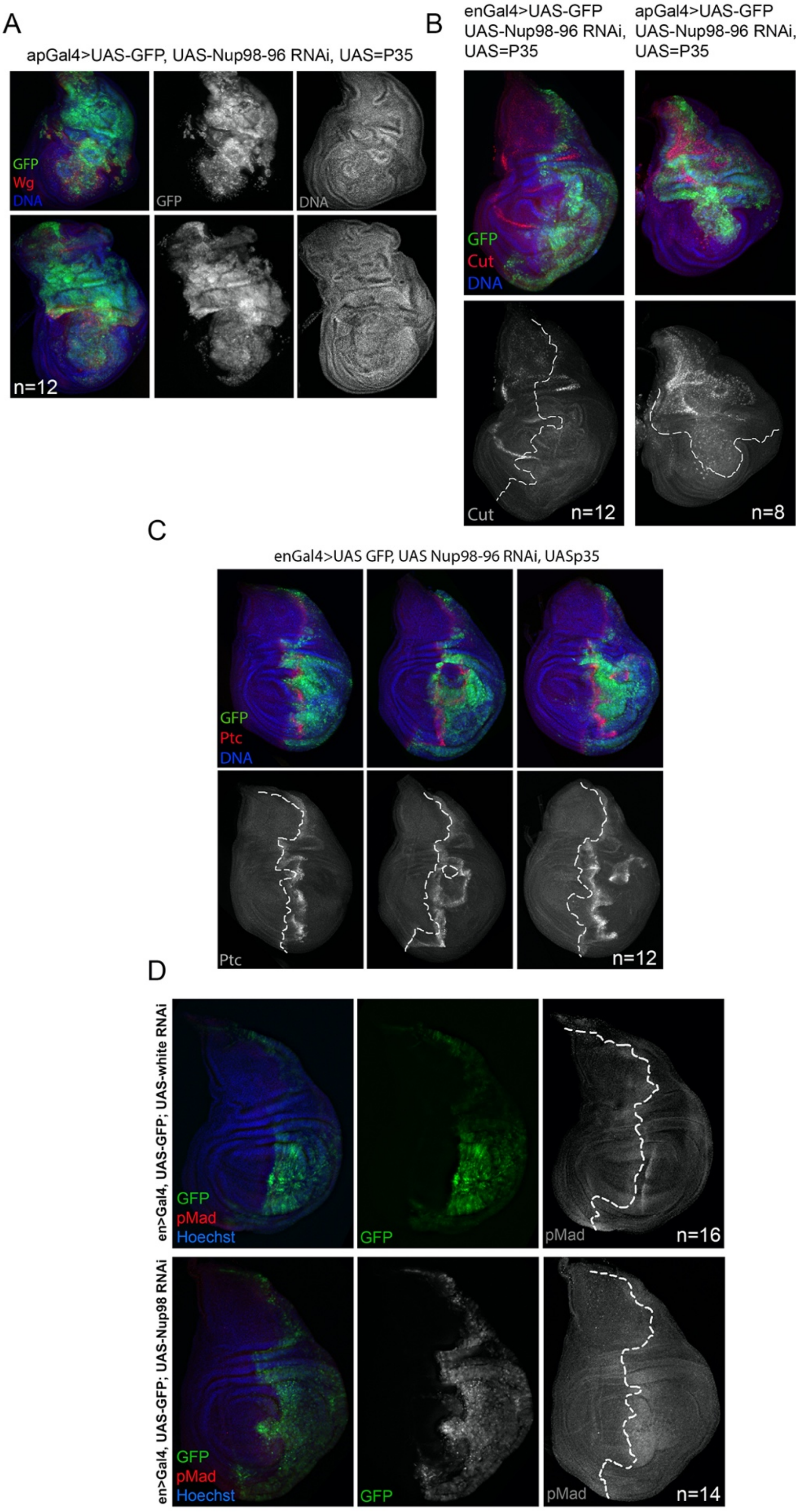

Disease Models & Mechanisms • Supplementary information

Fig S3. Knockdown of Nup98-96 leads to wing disc overgrowth and patterning defects consistent with apoptosis-induced proliferation.

(A) Expression of *Nup98-96 RNAi* in the dorsal compartment with *UAS-P35* for 5d (using *ap-Gal4, UAS-GFP/ UAS-P35; tub-Gal80^{TS}*) leads to tissue folding, overgrowth and invasion across the D-V boundary as well as ectopic Wg expression.

(B) Expression of *Nup98-96 RNAi + P35* in the posterior compartment (with *en-Gal4*, left) or dorsal compartment (with *ap-Gal4*, right) abolishes Cut expression at the D-V boundary.

(C) Expression of *Nup98-96 RNAi* in the posterior compartment with *UAS-P35* for 4d (using *en-Gal4, UAS-GFP/ UAS-P35; tub-Gal80^{TS}*) leads to tissue folding and invasion at the A-P boundary as well as ectopic Ptc expression demonstrating mis-patterning.

(D) Normal pMad staining is shown (top) for *en-Gal4, UAS-GFP; tub-Gal80^{TS}* driving *white* RNAi. (bottom) *Nup98-96 RNAi* expression driven by *en-Gal4, UAS-GFP; tub-Gal80^{TS}* leads to broad pMad staining in the posterior compartment indicating mis-patterning.

Disease Models & Mechanisms • Supplementary information

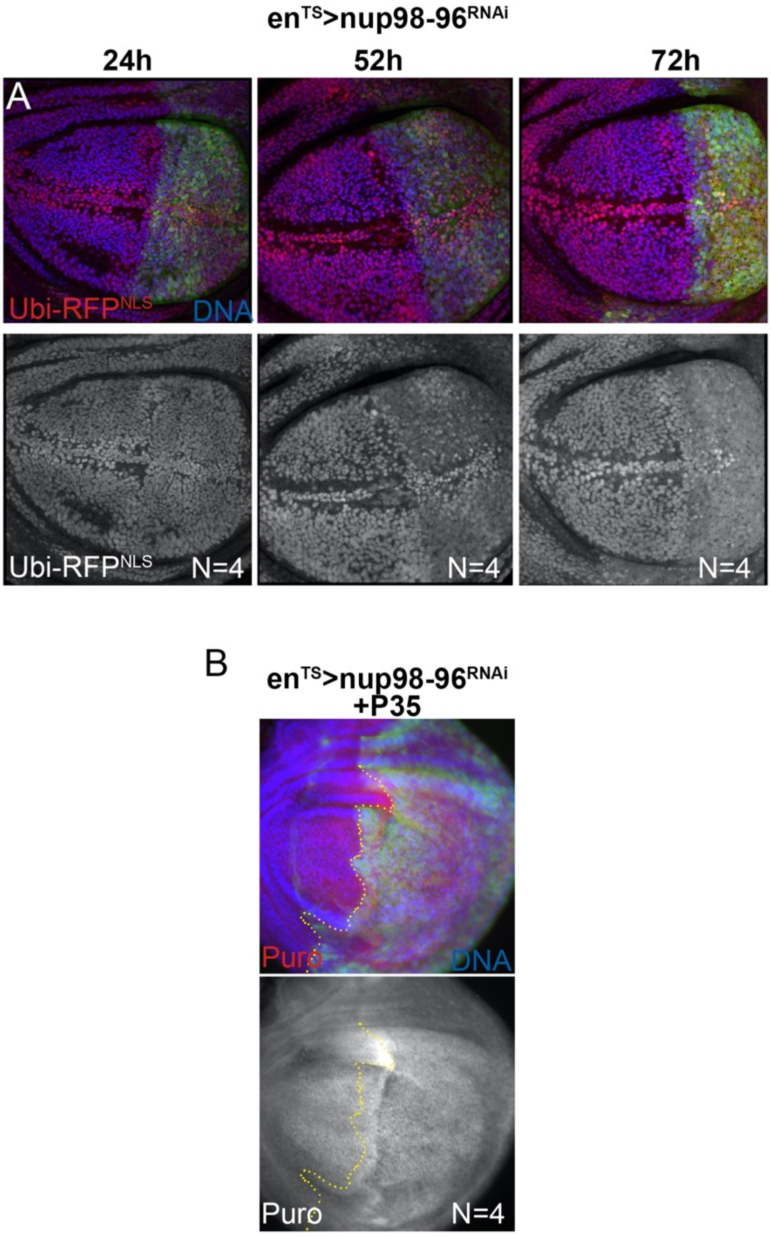

Fig. S4. Knockdown of Nup98-96 disrupts nucleo-cytoplasmic localization and reduces protein synthesis independent of apoptosis.

(A) *Nup 98-96 RNAi* was expressed in the posterior compartment using *en-Gal4, UAS-GFP; tub-Gal80^{TS}* in a background expressing *Ubiquitin* promoter driven-*RFP* with a nuclear localization signal (*Ubi-RFP^{NLS}*) at 27°C to minimize cell lethality. By 52h of Nup98-96 knockdown, nuclear localization of RFP is visibly disrupted. By 72h of knockdown nuclear localization of *Ubi*-RFP^{NLS} is disrupted.

(B) Co-expression of *UAS-P35* with *Nup 98-96 RNAi* did not rescue the reduction in protein synthesis when Nup98-96 is compromised. Protein synthesis was assayed by puro-labeling after 20h (n=4) and 40h (n=6) of *Nup98-96* knockdown. The data from 20h is shown. This suggests the reduced proteins synthesis is not a consequence of apoptosis.

Disease Models & Mechanisms • Supplementary information

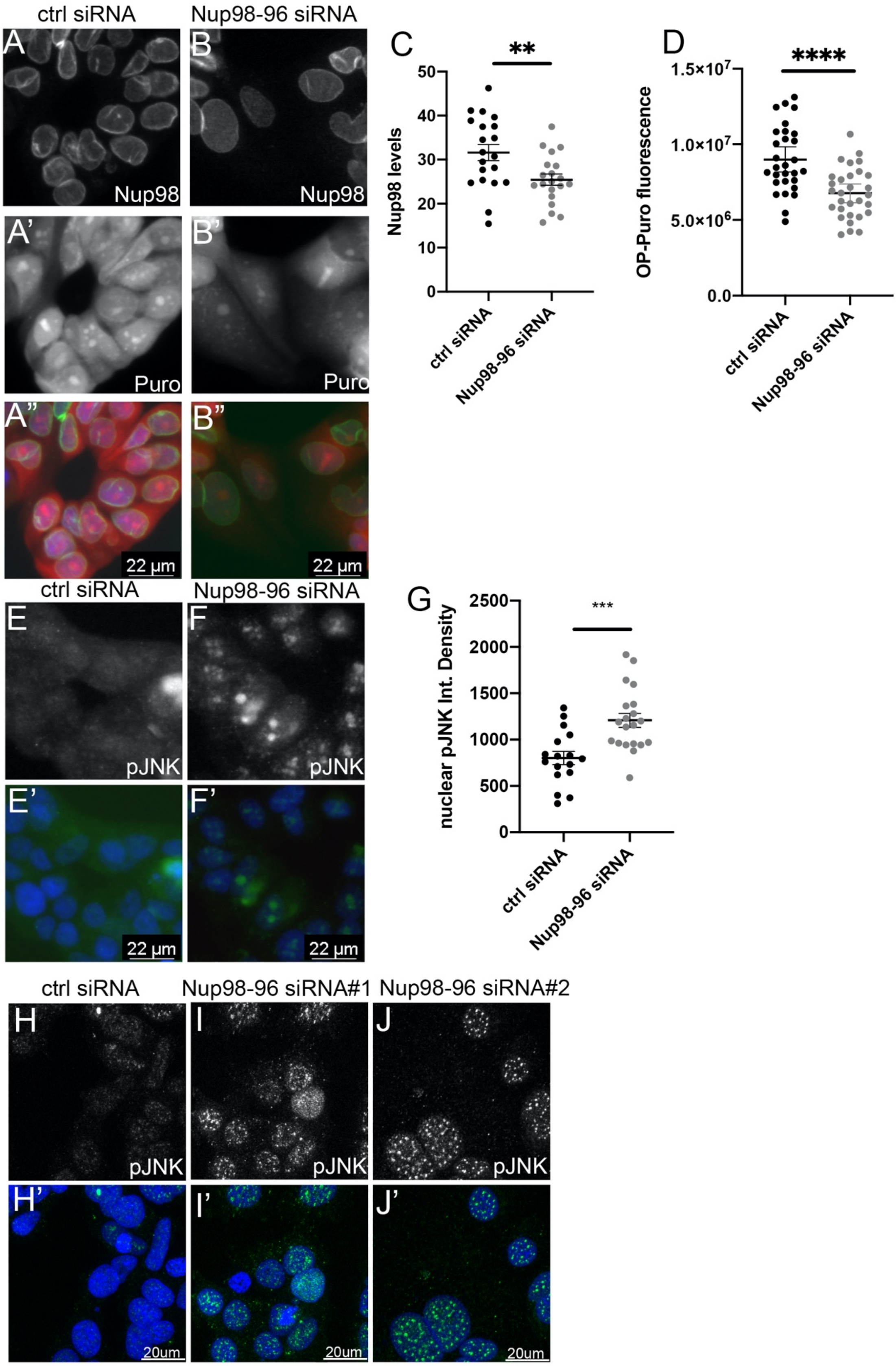

Disease Models & Mechanisms • Supplementary information

Fig S5. **Knockdown of Nup98 in MCF7 cells leads to reduced protein synthesis and JNK phosphorylation.**
(A-B, E-F, H-J) MCF7 cells were treated with small interfering (si) RNAs for 72h and cells were labeled with puro (A'-B') or fixed and stained with anti-Nup98 antibody (A-B), or phospho-JNK (E-J). Control siRNA (Ctrl) is a scrambled siRNA. Nup98 siRNA reduces Nup98 levels (C) as well as reduced protein synthesis (D) and increases pJNK labeling (G). Quantifications of fluorescence were performed on individual cells from at least two experiments. Three independent siRNAs were individually tested with siRNAs #1 and #2 being most effective on MCF7. ****$P<0.0001$, ***$P<0.001$, **$P<0.01$, *$P<0.05$ by unpaired t-tests, (G) uses Welch's correction for unequal sample size. Plots of individual biological replicates include mean±s.e.m.

Table S1. Excel file containing statistically significant gene expression changes >0.5 $\log_2$fold change in Nup98-96 knockdown L3 wing discs by RNA seq. Sheet 2 lists genes that overlap between changes in Nup98-96 knockdown and a Ras^{V12}, *scrib* invasive *Drosophila* tumor model.

Click here to download Table S1

Table S2. Excel file with the list of genes that overlap in Nup98-96 knockdown L3 wing discs and a wounding/regeneration program (Khan et al., 2017).

Click here to download Table S2

Table S3. Excel file with the list of genes that overlap in Nup98-96 knockdown L3 wing discs and a "loser" gene expression program (Kucinski et al., 2017).

Click here to download Table S3

Table S4. Excel file with the list of genes that overlap in Nup98-96 knockdown L3 wing discs and Xrp1 targets (Lee et al., 2018).

Click here to download Table S4

Table S5. Excel file with the list of genes that overlap in Nup98-96 knockdown L3 wing discs and Nup98 ChIP seq assays (Pascual-Garcia et al., 2017) and Nup98 alterations with RNAseq in S2 cells (Kalverda et al., 2010).

Click here to download Table S5

Disease Models & Mechanisms • Supplementary information

SUPPLEMENTARY MATERIALS AND METHODS

Fly stocks used

UAS-Nup98-96 RNAi (TRiP BL28562, VDRC lines, KK100388 and GD6897)

UAS-white RNAi (TRiP BL35573)

UAS-Dronc RNAi (TRiP BL 32963)

UAS-Drice RNAi (TRiP BL 32403)

UAS-Nmd3 RNAi (VDRC105619 and VDRC46166)

UAS-CG4364 RNAi (VDRC27607)

GMR-Gal4, UAS-CycE(I); GMR-P35 from H. Richardson

Nup98-96-GFP (VDRC 318656 FlyFos collection)

UAS-Nup98-96 cDNA(2M), UAS-Nup98 cDNA (3M), UAS-Nup98 cDNA (myc2F), UAS-Nup96 cDNA (myc7M), UAS-Nup96 cDNA (myc8M) all from C. Schulz and M. Capelson.

en^{TS} is w; *en-Gal4,UAS-GFP; tub-Gal80TS/TM6B* from Buttitta et al. (2007)

ap^{TS} is w; *ap-Gal4,UAS-GFP; tub-Gal80TS/TM6B* from Buttitta et al. (2007)

en^{TS} *RFP is w; en-Gal4,UAS-*RFP_{NLS}*; tub-Gal80TS/TM6B*

UAS-Bsk^{DN} (on III mutated in kinase domain) and puc^{e69}*-LacZ* provided by C. Collins.

UAS-P35 on X (BL6298)

RpS20-GFP (Kyoto 109696 w^{1118}; PBacRpS20^{KM0175} / TM2)

RpL5-GFP (Kyoto 109767 w^{1118}; PBac RpL5^{KM0174} / SM6a and Kyoto 109768 w^{1118}; PBac RpL5^{KM0163})

RpL10Ab-YFP (Kyoto 115462 w^{1118}; PBac RpL10Ab^{CPTI003957})

Ubi-RFP_{NLS}: (derived from BL35496)

y,w,hs-flp12 (derived from BL1929)

w; act>stop>Gal4, UAS-GFP_{NLS}*; UAS-P35* from Neufeld et al. (1998)

Antibodies

Mouse anti-PH3 Cell Signaling 9707 1:1000

Rabbit anti-PH3 Millipore 06-570 1:2000

Rabbit anti-Dcp1 Cell Signaling 9578 1:100

Disease Models & Mechanisms • Supplementary information

Rabbit anti-pJNK Promega v7931 1:100 (for *Drosophila*, used slightly younger pre-wandering larvae due to high peripodial signal in later larvae)

Rabbit anti-pSmad Cell Signaling 9516 1:50 (dissection must be performed on ice)

Rabbit anti-GFP Invitrogen A11122 1:1000 (for co-labeling GFP with EdU)

Mouse anti-cut DSHB 2B10 1:100

Mouse anti-lamin Dm0 DSHB ADL67.10 1:100

Mouse anti-Wg DSHB 4D4 1:100

Rabbit anti-Vg (1:200) via G. Schubiger, from S. Carroll

Mouse anti-Patched (1:200) via G. Schubiger, from T. Kornberg

Rabbit anti-Human Nup98 Cell Signaling C39A3 Rabbit used 1:500 for Western and 1:100 for immunofluorescence

Disease Models & Mechanisms • Supplementary information
